# ATP6V1B2 alleviates hepatic steatosis by promoting lysosomal acidification in hepatocytes

**DOI:** 10.1038/s41420-026-03052-8

**Published:** 2026-03-24

**Authors:** Ruizi Xu, Fuji Yang, Zhuan Zhang, Fang Cheng, Shihui Li, Yongmin Yan, Yanan Wang, Jing Zhou

**Affiliations:** 1 Department of Laboratory Medicine, Wujin Hospital Affiliated with Jiangsu University (Wujin Clinical College of Xuzhou Medical University), Changzhou, China; 2 Changzhou Key Laboratory of Exosome Basics and Translational Applications, Wujin Hospital Affiliated with Jiangsu University, Changzhou, China; 3 Department of Clinical Laboratory, The Affiliated Suzhou Hospital of Nanjing Medical University, Suzhou Municipal Hospital, Gusu School, Nanjing Medical University, Suzhou, Jiangsu China

**Keywords:** Macroautophagy, Lipidomics

## Abstract

Metabolic dysfunction-associated steatotic liver disease (MASLD) is a common metabolic disorder characterized by the abnormal accumulation of fat in the liver. ATP6V1B2, an essential subunit of the vacuolar ATPase (V-ATPase) complex, plays a pivotal role in its function and assembly. Despite its importance, the regulatory role of ATP6V1B2 and its molecular mechanism in MASLD progression remain poorly understood. In this study, we observed a significant reduction in ATP6V1B2 expression in the serum of MASLD patients. Experimental results demonstrated that inhibiting ATP6V1B2 expression in liver cells led to increased lipid accumulation, aggravated oxidative stress, upregulation of fatty acid synthase (FASN), and impaired autophagic activity. Further investigation revealed that ATP6V1B2 promotes the lysosomal degradation of FASN by maintaining the acidic environment of lysosomes, thereby playing a crucial role in lipid metabolism regulation. These findings uncover the critical mechanism by which ATP6V1B2 contributes to MASLD development and suggest that restoring its function could offer novel therapeutic strategies for treating this condition.

## Introduction

Metabolic dysfunction-associated steatotic liver disease (MASLD), previously referred to as non-alcoholic fatty liver disease (NAFLD), is a metabolic disorder closely associated with hepatic steatosis. It affects approximately one-quarter of the global population, rendering it the most prevalent chronic liver disease and a significant public health concern [1, 2]. Research has demonstrated that the onset and progression of MASLD are intricately linked to lipid accumulation, oxidative stress, and lipotoxicity [3]. The exacerbation of lipid deposition not only impairs the structure and function of the liver but also intensifies oxidative stress and endoplasmic reticulum (ER) stress through a series of complex biological mechanisms. Additionally, it promotes inflammation, inhibits lysosomal function, and hinders cellular autophagy [4]. Currently, our understanding of the biological mechanisms and clinical research related to liver steatosis remains relatively limited, underscoring the urgent need for further investigation.

Vacuolar ATPase (V-ATPase) is a multi-subunit protein complex that is expressed in nearly all eukaryotic organisms. This complex is composed of a soluble V1 subcomplex and a membrane-bound V0 subcomplex [5]. V-ATPase regulates intracellular acid-base homeostasis by pumping protons, thereby influencing various cellular processes, including nutrient uptake, metabolism, and autophagy [6]. Recent studies have highlighted V-ATPase’s involvement in regulating lipid metabolism, with particular relevance to MASLD [7]. Dysfunction of V-ATPase accelerates lipid droplet formation, exacerbating lipotoxicity and oxidative stress in hepatocytes [8, 9]. The expression of the V-ATPase subunit ATP6V0A2 can effectively reduce excessive oxidative stress and the accumulation of lipid peroxidation [10], and its functional changes are closely linked to disorders of lipid homeostasis and lysosomal dysfunction [7, 11]. In the context of MASLD, V-ATPase is involved in maintaining lysosomal acidification, which is essential for lipid droplet degradation and the overall regulation of lipid homeostasis [12, 13]. Research has also indicated that defects in V-ATPase assembly or function lead to an imbalance in lipid droplet-lysosome interactions, a key mechanism in MASLD pathogenesis [14, 15]. Collectively, these studies suggest that functional defects in V-ATPase are a common underlying process in the liver and contribute to lipid deposition observed in these diseases.

ATP6V1B2 is a component of V-ATPase, encoding the B2 subunit of this enzyme complex. It represents one of the two isoforms of the B subunit in the V1 domain and is highly expressed in the brain and lysosomes [16]. Research indicates that the structural characteristics of ATP6V1B2 are closely linked to its function, particularly in the acidic intracellular environment, where it plays a crucial role. Reports suggest that the overexpression of V-ATPase B2 in pulmonary fibrosis can effectively maintain lysosomal activity, thereby countering excessive oxidative stress [17]. Furthermore, ATP6V1B2 has been implicated in regulating autophagic flux in follicular lymphoma (FL) [18]. However, whether ATP6V1B2 regulates lysosomal activity in lipotoxic hepatocytes remains unclear. Notably, lysosomal function is significantly impaired in MASLD patients compared to both the normal population and those with fatty liver disease [19]. Previous studies have established a close relationship between ATP6V1B2 and lysosomal function [20]. Additionally, the mechanisms through which ATP6V1B2 may influence lipid metabolism and autophagy in MASLD are currently unknown. Further investigation is needed to determine whether ATP6V1B2 could serve as a potential therapeutic target for MASLD.

This study focuses on ATP6V1B2, a key subunit of the V-ATPase, and investigates its potential role and underlying molecular mechanisms in the pathogenesis and progression of MASLD. We first compared the expression levels of ATP6V1B2 in the serum of MASLD patients with those in healthy controls and found that ATP6V1B2 was significantly downregulated in the serum of patients with MASLD. We further investigated the mechanism of action of ATP6V1B2 in MASLD. The research findings indicate that ATP6V1B2 regulates lipid metabolism, alleviates oxidative stress and ER stress, and promotes cellular autophagy. Specifically, ATP6V1B2 enhances lysosomal acidification, thereby facilitating lysosomal autophagy and promoting the degradation of fatty acid synthase (FASN), which subsequently inhibits hepatic lipid accumulation. These findings provide new insights into the role of V-ATPase subunits in MASLD and offer a theoretical basis for developing targeted interventions based on the underlying molecular mechanisms.

## Results

### ATP6V1B2 has diagnostic value in the serum of patients with MASLD

**Fig. 1:**
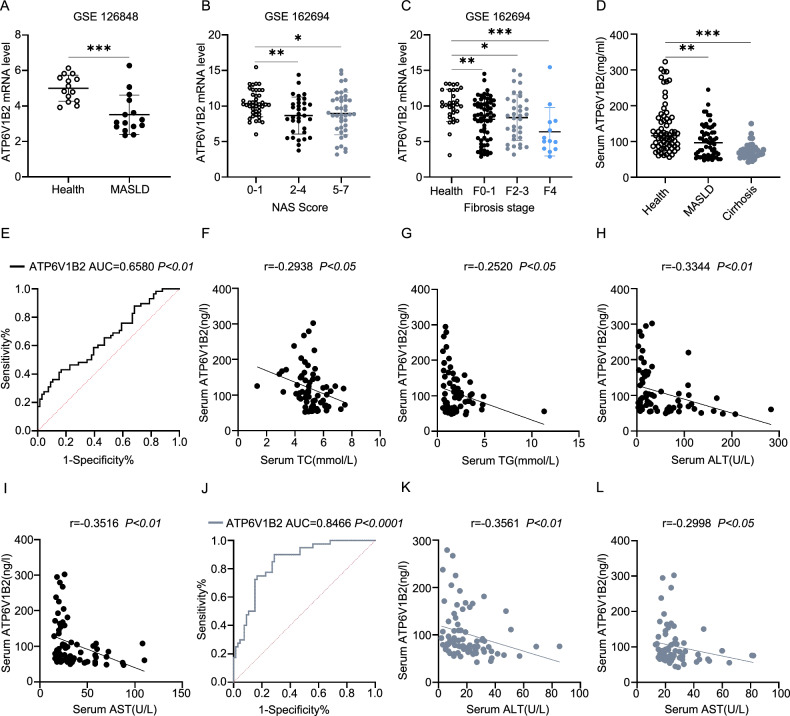
ATP6V1B2 has diagnostic value in the serum of patients with MASLD. **A** The relative levels of ATP6V1B2 were assessed in healthy individuals (*n* = 14) and patients with MASLD (*n* = 14) using data from the GSE126848 database. **B** The relative levels of ATP6V1B2 were evaluated across various MASH patients (n = 116) at different stages of the MASLD activity score (NAS) in the GSE162694 database. **C** The relative levels of ATP6V1B2 were assessed at various stages of liver fibrosis in patients with MASH (*n* = 143) from the GSE162694 database. **D** The relative levels of serum ATP6V1B2 were compared between healthy individuals (*n* = 66), patients diagnosed with MASLD (*n* = 58), and those with cirrhosis (*n* = 40). **E** ROC curves of serum ATP6V1B2 expression levels in healthy individuals (*n* = 66) and patients diagnosed with MASLD (*n* = 58). Serum **F** TC, **G** TG, **H** ALT, and **I** AST were analyzed for correlation with ATP6V1B2 in healthy individuals (*n* = 34) and MASLD patients (*n* = 34), respectively. **J** ROC curves of serum ATP6V1B2 change levels in healthy individuals (*n* = 66) and patients diagnosed with cirrhosis (*n* = 40). **K** Serum ALT, ATP6V1B2 correlation analysis in healthy individuals (*n* = 33) and patients diagnosed with cirrhosis (*n* = 37). **L** Serum AST, ATP6V1B2 correlation analysis in healthy individuals (*n* = 35) and patients diagnosed with cirrhosis (*n* = 39). *P < 0.05; **P < 0.01; ***P < 0.001.

To investigate the dysregulation of ATP6V1B2 in the progression of MASLD, we conducted a gene expression analysis of ATP6V1B2 in MASLD patients utilizing publicly available standardized datasets from the Gene Expression Omnibus (GEO) database. Our analysis of the GEO datasets GSE126848 and GSE162694 revealed that ATP6V1B2 expression in the liver tissue of MASLD patients was significantly lower compared to that of normal subjects (Fig. 1A). In patients with Metabolic Dysfunction-Associated Steatohepatitis (MASH), the expression level of ATP6V1B2 is negatively correlated with the NAFLD Activity Score (NAS) (Fig. 1B). A more advanced stage of liver fibrosis was further associated with a marked decline in ATP6V1B2 expression (Fig. 1C). We subsequently collected serum samples from MASLD patients, liver cirrhosis patients, and healthy controls to assess ATP6V1B2 expression levels. The results showed that serum ATP6V1B2 levels were significantly lower in both MASLD and liver cirrhosis patients compared to healthy controls (Fig. 1D). The AUROC analysis demonstrated that serum ATP6V1B2 had a diagnostic efficiency of 0.658 for MASLD (Fig. 1E). Moreover, serum ATP6V1B2 showed moderate correlations with serum total cholesterol (TC) (r = -0.2638, *p* < 0.05), triglycerides (TG) (r = -0.252, *p* < 0.05), alanine aminotransferase (ALT) (r = -0.3344, *p* < 0.01), and aspartate aminotransferase (AST) (r = -0.3516, *p* < 0.01) (Fig. 1F-I). Similarly, the AUROC analysis demonstrated that serum ATP6V1B2 had a diagnostic efficiency of 0.8466 for cirrhosis (Fig. 1J). ATP6V1B2 in serum exhibited strong correlations with ALT (r = -0.3561, *p* < 0.01) and AST (r = -0.2998, *p* < 0.05) (Fig. 1K, L). These results suggest that serum ATP6V1B2 may serve as a diagnostic indicator for MASLD and is potentially associated with the disease process.

### ATP6V1B2 expression is downregulated in MASLD mice and lipotoxic hepatocytes

**Fig. 2:**
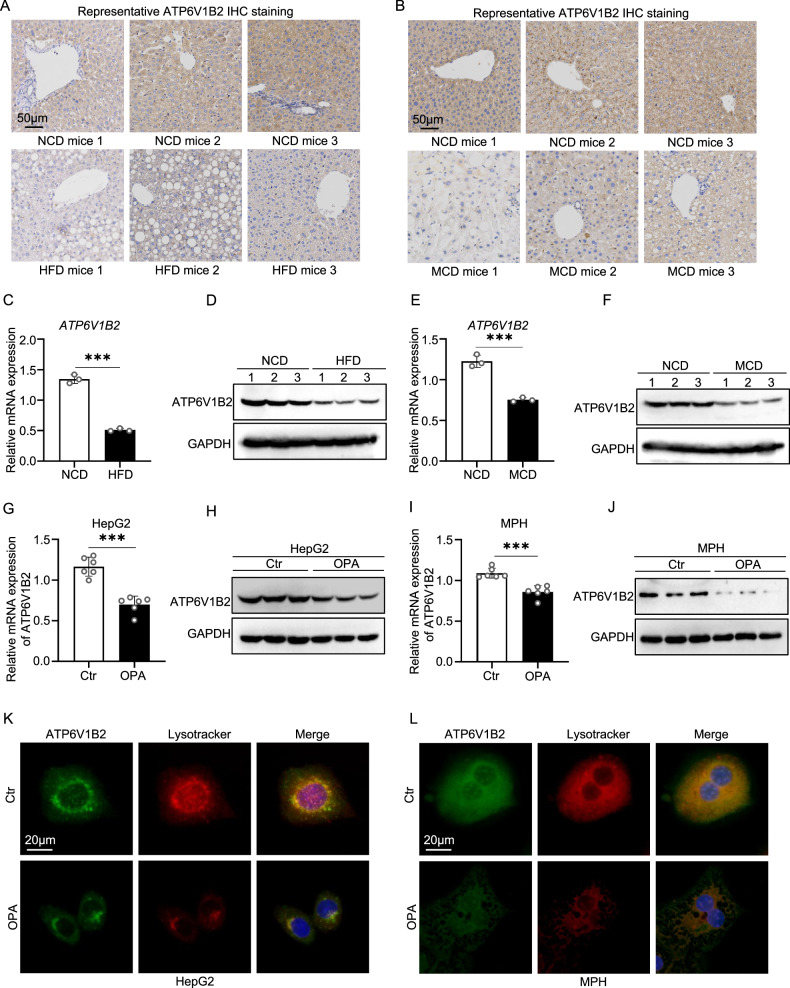
ATP6V1B2 expression is decreased in MASLD mice and lipotoxic hepatocytes. **A** Immunohistochemical staining of ATP6V1B2 in liver tissue of mice fed NCD or HFD for 14 weeks, *n* = 3. (Scale bar = 50 μm). **B** Immunohistochemical staining of ATP6V1B2 in liver tissue of mice fed NCD or MCD for 6 weeks, *n* = 3. (Scale bar = 50 μm). **C** mRNA and **D** protein expression levels in livers of NCD-fed or HFD-fed mice. ATP6V1B2 **E** mRNA and **F** protein expression levels in livers of NCD-fed or MCD-fed mice. ATP6V1B2 **G** mRNA and **H** protein expression levels in OPA-treated HepG2 cells. ATP6V1B2 **I** mRNA and **J** protein expression levels in OPA-treated MPH. LysoTracker Red staining and ATP6V1B2 immunofluorescence staining in OPA-treated **K** HepG2 cells and **L** MPH. (Scale bar = 20 μm). All data were expressed as the means ± SD of at least 3 independent experiments, *P < 0.05; **P < 0.01; ***P < 0.001.

To elucidate the role of ATP6V1B2 in MASLD, we established two mouse models of hepatic steatosis through high-fat diet (HFD) and methionine-choline-deficient diet (MCD) feeding. Our objective was to investigate the changes in ATP6V1B2 expression within the liver tissues of MASLD mice. Immunohistochemical staining demonstrated that the intensity of ATP6V1B2 positivity was significantly lower in the livers of both HFD diet-induced MASLD mice and MCD diet-induced MASH mice compared to those on a normal diet (Fig. 2A, B, and Supplementary Fig. 1A). Furthermore, both mRNA and protein expression levels of ATP6V1B2 in liver tissues were markedly reduced (Fig. 2C–F and Supplementary Fig. 1B, C).

Hepatic lipotoxicity, resulting from the accumulation of excess free fatty acids, is a crucial driver of MASLD. In reference to prior literature reports [21], we utilized a mixture of palmitic acid (PA) and oleic acid (OA), known as OPA, to establish an in vitro cell model of hepatocyte steatosis. HepG2 cells and mouse primary hepatocytes (MPH) were treated with 1 mM OPA for 24 hours. Nile red and Bodipy staining demonstrated that OPA treatment significantly induced lipid deposition in both HepG2 and MPH cells (Supplementary Fig. 1D, E). We first assessed the expression levels of ATP6V1B2 in MPH and HepG2 cells exposed to OPA. The results indicated that the mRNA and cellular protein expression levels of ATP6V1B2 in these two cell types were significantly reduced following OPA treatment (Fig. 2G-J and Supplementary Fig. 1F, G). Further analysis revealed a significant decrease in the expression of ATP6V1B2 in the cytoplasm of hepatocytes following OPA treatment, accompanied by a reduction in lysosomal activity. Additionally, a colocalization of ATP6V1B2 with lysosomes was observed (Fig. 2K, L and supplementary Fig. 1H, I).

To investigate how OPA affects the downregulation of ATP6V1B2 expression, we examined the mTOR signaling pathway, based on related literature indicating that lipid overload impacts this pathway [22]. The results showed that in HepG2 cells treated with OPA, the expression levels of mTOR and phosphorylated mTOR (p-mTOR) proteins significantly increased, while the expression of TFEB protein decreased (Supplementary Fig. 2 A). Concurrently, the nuclear translocation of TFEB was inhibited (Supplementary Fig. 2B). Given that TFEB is a major regulator of lysosome biogenesis, our findings further suggest that lysosomal impairment may result from TFEB dysfunction induced by lipotoxicity in the liver, ultimately leading to the reduced expression of the lysosome-related gene ATP6V1B2.

In summary, these findings suggest that the downregulation of ATP6V1B2 expression in the liver tissues of mice with hepatic steatosis, as well as in lipotoxic hepatocytes, may be attributed to lipid overload in hepatocytes. This overload affects the mTOR-TFEB axis, which subsequently influences lysosomal activity and leads to a reduction in ATP6V1B2 expression.

### ATP6V1B2 deficiency exacerbates lipid accumulation and oxidative stress in HepG2 cells

**Fig. 3:**
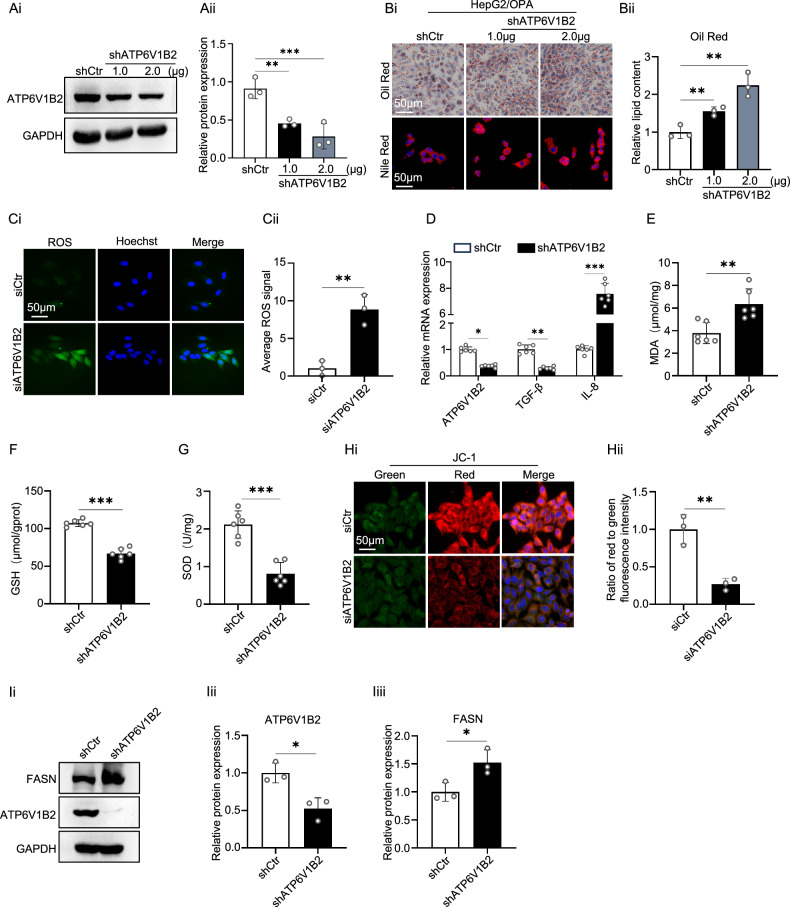
ATP6V1B2 deficiency exacerbates lipid deposition and oxidative stress in HepG2 cells. **A** HepG2 cells transfected with control plasmid (shCtr) or shATP6V1B2 plasmid (1 µg and 2 µg) were analyzed by Western blot for protein expression and quantified for ATP6V1B2 expression. **B** Nile red staining, oil red O staining and quantification of intracellular lipid droplets in 1.0 mM OPA-treated HepG2 cells transfected with shCtr or shATP6V1B2. (Scale bar = 50 μm). **C** ROS staining was used to assess ROS levels in HepG2 cells transfected with siCtr or siATP6V1B2. (Scale bar, 50 µm) **D** Quantification of TGF-β and IL-8 mRNA in HepG2-shCtr and HepG2-shATP6V1B2 cells. **E** MDA levels of HepG2-shCtr and HepG2-shATP6V1B2 cells. **F** GSH levels in HepG2-shCtr and HepG2-shATP6V1B2 cells. **G** SOD levels in HepG2-shCtr and HepG2-shATP6V1B2 cells. **H** JC-1 staining to detect mitochondrial membrane potential in HepG2 cells transfected with siCtr or siATP6V1B2. (Scale bar, 50 µm). **I** Western blot detection and quantification of FASN expression in HepG2-shCtr and HepG2-shATP6V1B2 cells. All data were expressed as the means ± SD of at least 3 independent experiments, * *P* < 0.05; **P < 0.01; ***P < 0.001.

To investigate the effect of ATP6V1B2 on hepatocyte steatosis, we first constructed a knockdown plasmid and siRNA targeting ATP6V1B2. Subsequently, we established HepG2 cells with stable ATP6V1B2 knockdown through recombinant ATP6V1B2-shRNA lentiviral infection and verified the knockdown efficiency (Supplementary Fig. 3A, B). The transfection of HepG2 cells with the shATP6V1B2 plasmid resulted in a significant reduction in both protein expression and mRNA levels of ATP6V1B2 (Fig. 3A and Supplementary Fig. 3C). To assess the alterations in lipid accumulation following ATP6V1B2 knockdown, we performed Nile red and oil red O staining concurrently. The findings indicated that lipid accumulation in HepG2 cells increased after the knockdown of ATP6V1B2 in lipotoxic hepatocytes (Fig. 3B).

Hepatic lipid accumulation affects multiple sources of reactive oxygen species (ROS) generation, including mitochondria, the ER, and lysosomes. The increased production of ROS leads to oxidative stress, inflammation, and alterations in the expression and activity of crucial enzymes involved in lipid metabolism [23]. To further investigate the role of ATP6V1B2 in hepatocyte oxidative stress, we performed a ROS assay following the silencing of ATP6V1B2 expression. Our findings revealed that the intracellular levels of ROS increased in parallel with the decrease in ATP6V1B2 expression (Fig. 3C). Additionally, quantitative reverse transcription polymerase chain reaction (qRT-PCR) results demonstrated that downregulation of ATP6V1B2 expression was associated with a reduction in TGF-β expression and a corresponding increase in the inflammatory factor IL-8 (Fig. 3D). Furthermore, malondialdehyde (MDA), a key marker of lipid peroxidation, exhibited a significant elevation (Fig. 3E). The levels of glutathione (GSH), an important indicator of oxidative stress, were found to decrease, as did the levels of the antioxidant enzyme superoxide dismutase (SOD) (Fig. 3F, G). Mitochondrial membrane potential is crucial for maintaining normal mitochondrial function. In this study, we assessed the changes in mitochondrial membrane potential in hepatocytes following the silencing of ATP6V1B2 using JC-1 staining. The experimental results indicated a significant reduction in the ratio of red-to-green fluorescence compared to the control group, indicating a disturbance in mitochondrial membrane potential (Fig. 3H). This disturbance negatively affects normal cellular metabolism and autophagy. Collectively, these results suggest that the absence of ATP6V1B2 expression contributes to oxidative stress injury and exacerbates the inflammatory response in hepatocytes.

The knockdown of ATP6V1B2 resulted in the upregulation of ER stress markers, including GRP78, CHOP, PERK, IRE1, and XBP1 (Supplementary Fig. 3D, E), thereby activating the unfolded protein response (UPR) pathway. Previous studies have demonstrated that the IRE1α-XBP1 pathway is activated in lipotoxic hepatocytes [24], and that increased expression of XBP1 can enhance the promoter activity of the FASN gene, subsequently initiating lipogenesis in the liver [25]. Our examination of the effects of ATP6V1B2 knockdown on FASN revealed that protein levels of FASN were significantly elevated following ATP6V1B2 knockdown (Fig. 3I).

These comprehensive results indicate that the loss of ATP6V1B2 expression induces cellular oxidative stress, which leads to ER stress and the subsequent activation of the UPR. Furthermore, the activation of the IRE1α-XBP1 signaling pathway influences the expression of FASN in hepatocytes.

### ATP6V1B2 alleviates lipid deposition and oxidative stress in lipotoxic HepG2 cells

**Fig. 4:**
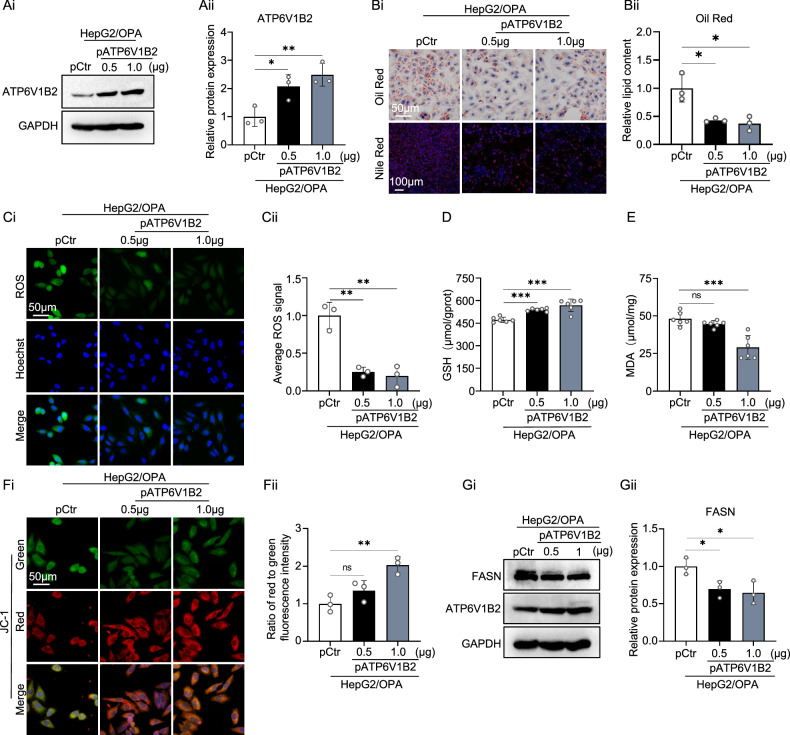
ATP6V1B2 inhibits OPA-induced lipid deposition and oxidative stress in HepG2 cells. **A** Western blot detection and quantification of protein expression in OPA-treated HepG2 cells transfected with control plasmid (pCtr) or pATP6V1B2 plasmid (0.5 µg or 1 µg). **B** HepG2 cells, transfected with either shCtr or shATP6V1B2, were treated with 1.0 mM OPA and subsequently subjected to Nile red staining (scale bar = 100 μm), oil red O staining, and quantitative analysis of lipid droplets (scale bar = 50 μm). **C** ROS staining to detect reactive oxygen species levels in OPA-treated HepG2 cells transfected with pCtr or pATP6V1B2. (Scale bar = 50 µm). **D** GSH and **E** MDA levels in OPA-treated HepG2 cells transfected with pCtr or pATP6V1B2. **F** JC-1 staining to detect mitochondrial membrane potential in OPA-treated HepG2 cells transfected with pCtr or pATP6V1B2. (Scale bar = 50 µm). **G** Western blot detection of FASN expression in OPA-treated HepG2 transfected with pCtr or pATP6V1B2. All data were expressed as the means ± SD of at least 3 independent experiments, * P < 0.05; **P < 0.01; ***P < 0.001.

We generated ATP6V1B2-overexpressing HepG2 cells using a plasmid (pATP6V1B2). Western blot analysis confirmed the successful overexpression of ATP6V1B2 in HepG2 cells (Fig. 4A). Staining with Nile Red and Oil Red O indicated that ATP6V1B2 overexpression significantly reduced lipid droplet accumulation in OPA-stimulated hepatocytes (Fig. 4B). OPA treatment induced oxidative damage in the cells; however, after overexpression of ATP6V1B2 in lipotoxic hepatocytes, attenuated ROS fluorescence was observed, suggesting enhanced ROS scavenging capacity of the cells (Fig. 4C). With the increase in ATP6V1B2 expression, qRT-PCR results showed an upregulation of TGF-β expression, while the expression of the inflammatory factor IL-8 was suppressed (Supplementary Fig. 3A). Additionally, cellular GSH levels increased, and MDA levels decreased significantly (Fig. 4D, E). Compared to the control group, lipotoxic hepatocytes overexpressing ATP6V1B2 exhibited an elevated mitochondrial membrane potential, effectively restoring mitochondrial homeostasis (Fig. 4F).

Lipotoxicity induces ER stress, while the overexpression of ATP6V1B2 reduces the expression levels of ER stress markers such as GRP78, CHOP, PERK, IRE1, and XBP1 in lipotoxic hepatocytes (Supplementary Fig. 4B). This finding suggests a degree of alleviation of ER stress. Additionally, the overexpression of ATP6V1B2 in lipotoxic hepatocytes leads to a decrease in the expression of FASN (Fig. 4A, G).

Collectively, these data indicate that ATP6V1B2 may play a role in regulating lipid metabolism and alleviating hepatic steatosis, potentially through mechanisms involving the reduction of cellular oxidative stress and the inhibition of de novo lipid synthesis.

### ATP6V1B2 regulates lysosomal acidification and promotes cellular autophagy in lipotoxic HepG2 cells

**Fig. 5:**
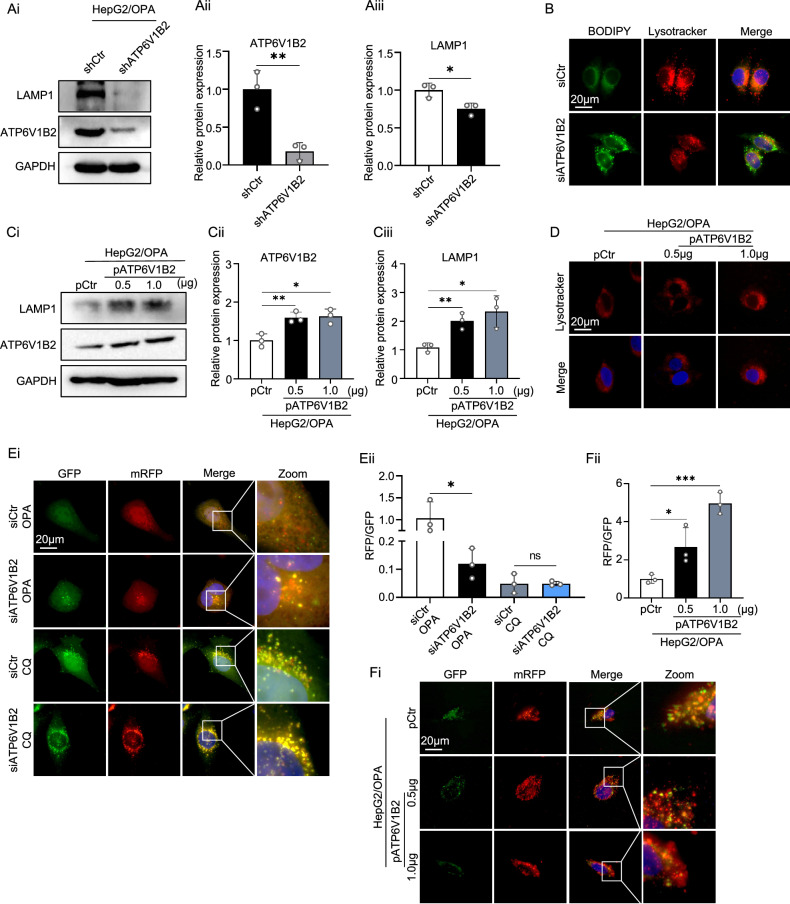
ATP6V1B2 acidifies lysosomes to promote cellular autophagy. **A** Western blot detection and quantification of LAMP1 expression in OPA-treated HepG2-shCtr and HepG2-shATP6V1B2 cells. **B** LysoTracker Red and Bodipy probe fluorescence staining was observed in HepG2 cells transfected with siCtr and siATP6V1B2. (Scale bar = 20 µm). **C** Western blot detection and quantification of LAMP1 expression in OPA-treated HepG2 transfected with pCtr or pATP6V1B2. **D** LysoTracker Red staining was observed in OPA-treated HepG2 cells transfected with pCtr or pATP6V1B2. (Scale bar = 20 µm). **E** HepG2 transfected with autophagy double-labeled lentivirus mRFP-GFP-LC3B in HepG2 transfected with siCtr and siATP6V1B2 treated with OPA or CQ were microscopically excited at 555 nm (red) and 493 nm (green), respectively. (Scale bar = 20 µm). **F** Transfection of pCtr or pATP6V1B2 in OPA-treated HepG2 transduced with autophagic double-labeled lentivirus mRFP-GFP-LC3B, microscopically excited at 555 nm (red) and 493 nm (green), respectively. (Scale bar = 20 µm). All data were expressed as the means ± SD of at least 3 independent experiments, *P < 0.05; **P < 0.01; ***P < 0.001.

V-ATPase regulates lysosomal acidification and is closely associated with lysosomal function [26]. In OPA-treated HepG2 cells with knockdown of ATP6V1B2, western blot results showed decreased expression of the lysosomal membrane surface marker LAMP1 (Fig. 5A). The lysosome-specific probe, LysoTracker, was employed to detect HepG2 cells. This probe selectively enters acidic lysosomes and serves as a marker for the acidic environment, indirectly reflecting lysosomal functional status. In cells with silenced ATP6V1B2, the fluorescence of the red probe was significantly reduced, indicating diminished lysosomal acidity and activity; lipid droplet staining also revealed a greater accumulation of lipid droplets within these cells (Fig. 5B). In HepG2 cells treated with the lysosomal enzyme fluorescent substrate DQ Red BSA, a significant reduction in red fluorescence was observed in cells with ATP6V1B2 knockdown, indicating a decrease in lysosomal proteolytic activity (Supplementary Fig. 4C). Conversely, the overexpression of ATP6V1B2 in lipotoxic hepatocytes enhanced lysosomal activity and restored lysosomal acidity (Fig. 5C, D).

V-ATPase is essential for lysosome-mediated autophagy, which is involved in the degradation of macromolecules, including lipids, proteins, and sugars [27]. To investigate the impact of ATP6V1B2 deficiency on autophagy in hepatocytes, we used the mRFP-GFP-LC3B reporter system to visualize autophagic flux. In this system, GFP fluorescence decreases in acidic environments, while mRFP fluorescence remains stable, allowing us to differentiate between autophagosomes (yellow) and autolysosomes (red). The results showed that in ATP6V1B2-silenced HepG2 cells, impaired lysosomal acidification led to increased GFP signal, resulting in more yellow dots. This suggests that autophagosomes were unable to fuse properly with lysosomes, hindering the degradative function of autolysosomes. Additionally, the inhibition of lysosomal function with chloroquine (CQ) also suppressed autolysosome formation (Fig. 5E). Overexpression of ATP6V1B2 in lipotoxic hepatocytes promoted the conversion of autophagosomes to autolysosomes, increasing the number of autolysosomes and enhancing autophagic flux efficiency (Fig. 5F).

### ATP6V1B2 promotes cellular lysosomal degradation of FASN

**Fig. 6:**
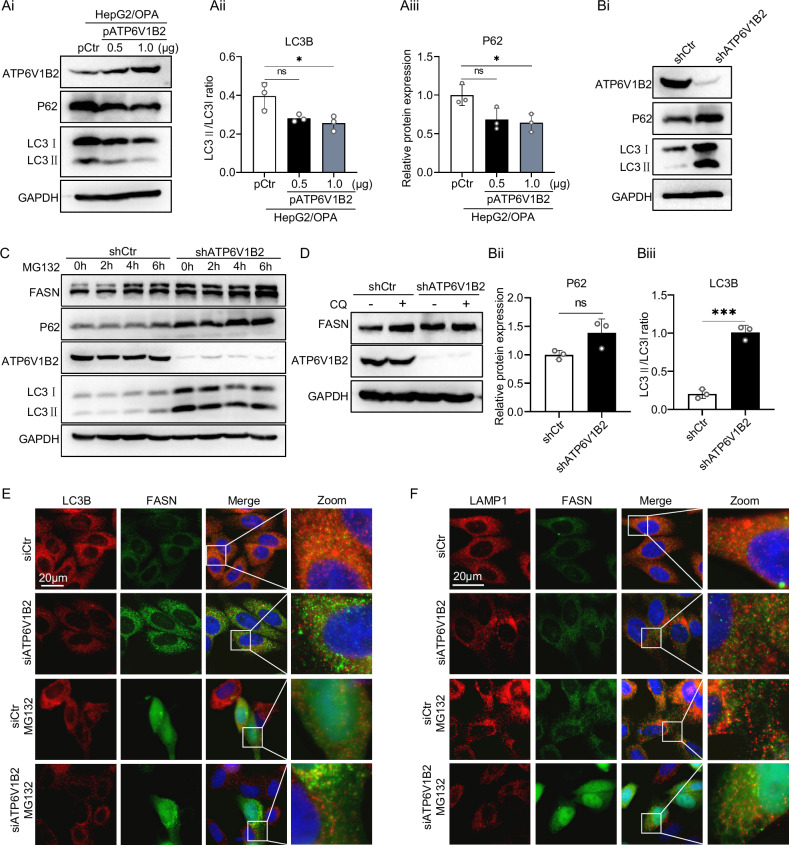
ATP6V1B2 promotes autophagic degradation of FASN protein. **A** Western blot detection of P62, LC3B expression, and quantification in OPA-treated HepG2 transfected with pCtr or pATP6V1B2. **B** Western blot detection of P62, LC3B protein expression, and quantification in HepG2-shCtr and HepG2-shATP6V1B2 cells. **C** Western blot to detect the expression of FASN, P62, and LC3B proteins in MG132 (20 µM)-treated HepG2-shCtr and HepG2-shATP6V1B2 cells. **D** Western blot detection of FASN protein expression in CQ (20 µM)-treated HepG2-shCtr and HepG2-shATP6V1B2 cells. **E** Immunofluorescence detection of FASN and LC3B expression in MG132 (20 µM)-treated HepG2 cells transfected with siCtr and siATP6V1B2. (Scale bar = 20 µm). **F** Co-localization of FASN and LAMP1 in HepG2 cells transfected with siCtr and siATP6V1B2 detected by LysoTracker Red and immunofluorescence. (Scale bar = 20 µm). All data were expressed as the means ± SD of at least 3 independent experiments, *P < 0.05; **P < 0.01; ***P < 0.001.

To further elucidate the role of ATP6V1B2 in autophagic flux, we examined the protein levels of LC3-II and P62 through western blot analysis. Following the overexpression of ATP6V1B2 in lipotoxic hepatocytes, we observed a decrease in both LC3-II and P62 levels, indicating that ATP6V1B2 promotes autophagy (Fig. 6A). Conversely, in cells with ATP6V1B2 knockdown, both LC3-II and P62 levels increased (Fig. 6B), indicating a reduction in hepatocyte autophagic flux following ATP6V1B2 knockdown. Collectively, these results underscore the regulatory role of ATP6V1B2 in autophagy. Impaired expression of ATP6V1B2 can hinder the formation of autophagic lysosomes and obstruct autophagic flux, thereby leading to autophagic dysfunction.

Autophagy is responsible for the degradation of numerous intracellular components, including proteins [28]. Previous studies have indicated that damage to autophagy leads to increased expression of FASN, resulting in enhanced formation of lipid droplets and reduced degradation [29]. Consequently, we investigated whether ATP6V1B2 regulates the degradation of FASN. Initially, HepG2 cells expressing shCtr or shATP6V1B2 were treated with the proteasome inhibitor MG132 across a time gradient. Treatment with MG132 resulted in the accumulation of FASN in HepG2-shCtr cells, which persisted for up to 6 hours, indicating that FASN can be degraded via the proteasome pathway. However, in the presence of MG132, the concentration of FASN protein in HepG2-shATP6V1B2 cells was significantly higher than in the control group, suggesting the presence of a second, independent mechanism regulating FASN degradation (Fig. 6C). Next, we examined whether ATP6V1B2 influences the lysosomal degradation of FASN. After treatment with CQ to elevate lysosomal pH, FASN levels increased in HepG2 cells, but not in ATP6V1B2 knockout cells (Fig. 6D). Simultaneously, HepG2 cells were treated with MG132 to inhibit the proteasome degradation pathway. The colocalization of FASN and LC3B was significantly diminished in ATP6V1B2 knockdown cells (Fig. 6E), and the colocalization of FASN and LAMP1 was also markedly reduced (Fig. 6F). Collectively, these results suggest that ATP6V1B2 promotes the lysosomal degradation of FASN.

## Discussion

The course of MASLD is long and complex, making in-depth research into its mechanisms crucial. To investigate the role of ATP6V1B2 in MASLD, we conducted a case-control study within the Chinese population, revealing that serum concentrations of ATP6V1B2 were significantly reduced in patients with MASLD. Notably, ATP6V1B2 levels decreased progressively with increasing disease severity, demonstrating an inverse correlation with MASLD severity. This clinical observation offers a novel entry point for further research. Consistent with our clinical findings, we observed reduced transcript and protein levels of ATP6V1B2 in a lipotoxic hepatocyte model, suggesting that lipotoxicity induces a decrease in ATP6V1B2 expression in hepatocytes.

MASLD triggers ER stress and cell death as a result of lipid deposition. ER stress influences hepatic lipid metabolism [30]. Concurrently, ROS generated during lipid metabolism induce oxidative stress, exacerbate inflammatory responses, impair hepatocyte function, and promote the progressive deterioration of the disease [31–33]. It is important to consider strategies for inhibiting steatosis and inflammation, as these are critical measures for preventing the progressive deterioration of MASLD. Defects in V-ATPase subunits or associated proteins have been increasingly recognized as contributing factors in various lipid metabolism disorders. These defects play significant roles in oxidative stress, ER stress, and the UPR[8, 34]. ER stress can regulate lysosome biogenesis to maintain cellular homeostasis [35]. Evidence suggests that the overexpression of V-ATPase B2 enhances lysosomal activity in cells, thereby mitigating excessive oxidative stress and reducing pulmonary fibrosis [17]. The activity of V-ATPase is essential for protecting cells against endogenous oxidative stress [36]. Our results indicated that the expression level of FASN decreased, the level of the anti-inflammatory factor TGF-β decreased, and the expression level of the inflammatory factor IL-8 increased in ATP6V1B2 knockdown HepG2 cells. Furthermore, overexpression of ATP6V1B2 alleviated the oxidative stress, ER stress, and inflammatory response induced by hepatic lipotoxicity resulting from hepatocyte fat deposition. These findings underscore the role of ATP6V1B2 in MASLD and suggest a novel therapeutic strategy.

V-ATPase is a pH-sensitive proton pump that utilizes the energy derived from ATP hydrolysis to transport hydrogen ions into lysosomes, thereby establishing and maintaining an acidic environment within these organelles. This multi-subunit complex plays a crucial role in various cellular processes by regulating lysosomal acidification [6]. As a member of the V-ATPase family, mutations in ATP6V1B2 impair lysosomal V-ATPase activity, consequently affecting the degradation capacity of lysosomes [20]. Notably, the protein levels of V-ATPase subunits were significantly reduced in liver lysosomes isolated from mice fed a high-fat diet, which in turn disrupted lysosomal acidification [12].

Current evidence suggests that cholesterol and free fatty acids can promote the onset and progression of MASLD by abnormally activating the mTOR signaling pathway and inhibiting the TFEB-dependent autophagy-lysosome pathway [37]. This study found that lipid overload leads to lysosomal dysfunction by activating mTOR and inhibiting TFEB nuclear translocation, which may underlie the reduced expression of ATP6V1B2 in lipotoxic hepatocytes. Further experiments confirmed the presence of impaired lysosomal acidification, autophagic flux blockade, and lysosomal dysfunction in the lipotoxic hepatocyte model; these pathological changes may directly contribute to the disease process of MASLD. According to existing reports, lysosomal dysfunction and the blockade of autophagy flow caused by ATP6V1B2 dysregulation are closely associated with neuronal degeneration [38]. Interestingly, damage to autophagic degradation can be mitigated by enhancing the expression of V-ATPase B2, which in turn promotes autophagic flux [39]. In the context of metabolic diseases such as MASLD, the roles of lysosomal autophagy and V-ATPase have garnered significant attention. Our findings demonstrate that the loss of ATP6V1B2 impairs lysosomal acidification and disrupts its function, thereby exacerbating oxidative stress and fat accumulation in hepatocytes. Additionally, we provide evidence that increased expression of ATP6V1B2 in lipotoxic hepatocytes can enhance lysosomal activity and inhibit cellular lipid deposition. In summary, these findings elucidate the mechanism by which ATP6V1B2-mediated lysosomal acidification prevents intrahepatocyte fat accumulation in MASLD and underscore potential therapeutic targets for its management.

Disordered lipid metabolism is a critical factor in the pathogenesis of MASLD, where FASN, a key rate-limiting enzyme in de novo fatty acid synthesis, has been confirmed to play a significant role in promoting hepatic lipid deposition when its levels are abnormally elevated [40]. Recent studies indicate that reduced autophagic activity hinders the degradation of FASN, which serves as an autophagy substrate, leading to its upregulation [41]. However, the mechanisms by which the lysosomal autophagy pathway regulates FASN degradation in MASLD remain unclear. This study provides evidence that in lipotoxic hepatocytes, ATP6V1B2 significantly enhances FASN degradation and inhibits lipid deposition by activating the lysosome-dependent autophagy pathway. Furthermore, previous research has demonstrated that ER stress markedly increases the transcriptional expression of FASN via the IRE1α-XBP1 axis [42]. Consistent with this, we also observed that ATP6V1B2 knockdown induces ER stress, thereby activating the IRE1α-XBP1 signaling pathway and influencing the transcriptional expression levels of FASN in hepatocytes. These results suggest that ATP6V1B2 may regulate FASN expression through the IRE1α-XBP1-FASN signaling pathway. This study experimentally confirms that ATP6V1B2 can degrade FASN protein through the autophagy pathway, thereby providing a novel mechanism for the regulation of FASN protein levels. Notably, in addition to the demonstrated protein degradation effect, our preliminary analysis suggests that ATP6V1B2 may also be involved in regulating FASN gene transcription or mRNA stability (Supplementary Fig. 3F, 4D). Existing studies have reported that autophagy-related proteins and lysosomal functions can indirectly affect the expression of lipid metabolism-related genes through multiple signaling pathways [43, 44]. As a key subunit of V-ATPase, ATP6V1B2 plays a central role in maintaining lysosomal acidification and function; its dysfunction may impact downstream transcriptional regulatory networks. Therefore, we will further investigate the effect of ATP6V1B2 on FASN expression at the transcriptional level to provide a more comprehensive understanding of its role in metabolic homeostasis.

Nevertheless, this study also has several limitations. Our findings indicate that ATP6V1B2 can restore lysosomal acidification in lipotoxic hepatocytes and exhibits certain therapeutic effects. However, the therapeutic efficacy of ATP6V1B2 in MASLD mice remains uncertain. Restoring lysosomal acidification, reversing the autophagic flux in lipotoxic hepatocytes, and reducing lipid deposition in hepatocytes are critical strategies for treating MASLD. Some acidic nanoparticles that enhance lysosomal acidification have demonstrated efficacy in alleviating MASLD in mice [45]. Nonetheless, investigating whether we can directly enhance lysosomal function by targeting ATP6V1B2 presents significant research potential and could emerge as an important avenue for clinical treatment of MASLD in the future.

Our results demonstrate that the concentration of ATP6V1B2 in the serum of patients with MASLD and cirrhosis is lower than that in healthy individuals, and it is inversely correlated with the severity of MASLD and metabolic risk factors. ATP6V1B2 plays an essential role in autolysosome formation, thereby enabling cells to effectively eliminate damaged organelles and engulfed bacteria and limiting inflammation. Additionally, it regulates lysosomal activity to promote the autophagic degradation of FASN, which alleviates lipid accumulation in lipotoxic hepatocytes. Our findings also emphasize the potential of lysosome-targeted therapy as a therapeutic strategy for MASLD, highlighting its importance in MASLD research.

## Materials and methods

### Cell culture

The human hepatocellular carcinoma cell line HepG2 was obtained from Nanjing BioChannel Biotechnology Co., Ltd. It underwent correct STR identification and was confirmed to be free from mycoplasma contamination. HepG2 cells were cultured using DMEM high glucose medium (BCM005, Biochannel, China) supplemented with 10% fetal bovine serum (BC-SE-FBS07, Biochannel, China) and 1% penicillin /streptomycin (C0222, Beyotime, China). Mouse primary hepatocytes (MPH) were obtained from 6-8 weeks old C57BL/6 male mice. A standard two-step collagenase perfusion method was used to isolate mouse primary hepatocytes. An in vitro lipid deposition cell model was constructed using oleic acid (07501, Sigma, USA) and palmitic acid (P0500, Sigma, USA), dissolved in 5% BSA (BS114-100g, Biosharp, China) solution, a mixture was prepared at a concentration of 1.0 mM OPA (oleic acid: palmitic acid = 2:1), and cells were treated for 24 hours.

### Mouse model

The animals used in this study were male C57BL/6 mice to establish the MASLD model. The mice were maintained in an environment with a light/dark cycle of 12 hours, a temperature of 23 ± 2°C, and a relative humidity of 55 ± 5%. Experimental mice were randomly divided into 4 groups: normal diet group (*N* = 3), where mice were fed normal chow (NCD, 1010001, XIETONG BIO-ENGINEERING, China); and HFD diet group (*N* = 3), where mice were fed high-fat chow (HFD, H10060, HUAFUKANG Bioscience, China) for 14 weeks; and the MCD diet group (*N* = 3), fed methionine- and choline-deficient chow (MCD, MD12052, Medicience, China) for 6 weeks.

### Western blot

Cells or tissues were lysed on ice using RAPA (P0013B, Beyotime, China) containing 1% PMSF (BL507A, Biosharp, China), cells or tissues were lysed by sonication, and the protein supernatant was collected by centrifugation for 15 min, and the protein concentration was determined by the BCA (Mei5Bio) method after adding the Loading Buffer (BL502B, Biosharp, China) was mixed and heated at 95 °C for 10 min. Equal amounts of proteins were loaded and separated on 10% or 12% SDS-PAGE gels, and proteins were transferred to 0.22 μm PVDF membranes (ISEQ 00010, Merck Millipore) enclosed in 5% skimmed milk (S1013-90, ABiomol) for 2 hours. Primary antibodies and HRP-coupled secondary antibodies were incubated. An ECL detection system was used for signal detection. Band intensity was quantified by ImageJ. The primary and secondary antibodies used in this study are listed in Additional file 2: Supplementary Table 1.

### Immunofluorescence

Cells were fixed with 4% paraformaldehyde for 15 min, washed with PBS, and permeabilized with 0.1% Triton X-100 (P0096, Beyotime, China) for 10 min. after washing with PBS, the cells were closed with 5% BSA (BS114-100g, Biosharp, China) for 1 h. The cells were then incubated with primary antibody at 4 °C overnight. Subsequently, the samples were washed with PBS, and the fluorescent secondary antibody was incubated in a dark environment at room temperature for 1 h. Finally, the cells were stained with Hoechst 33342 staining solution (Beyotime, China) for 5 min. Images were captured by fluorescence microscopy (Olympus, Japan). The primary and secondary antibodies for immunofluorescence are provided in Additional file 2: Supplementary Table 1.

### Quantitative reverse transcription PCR

Total RNA was extracted from cells and mouse liver tissues using Trizol (15596026, Ambion, USA). One microgram of total RNA was reverse transcribed into cDNA using the HiScript III 1st Strand cDNA Synthesis Kit (R312-01, Vazyme, China). qRT-PCR was performed using 2× Rapid Taq Master Mix (P222-01, Vazyme, China) on the CFX96 Touch™ Real-Time PCR Detection System (Bio-Rad, USA). Relative gene expression was determined using the 2-ΔΔCT method, normalized to the β-actin gene. PCR primer sequences are shown in Additional file 2: Supplementary Table 2.

### Patient clinical samples

We collected discarded human serum samples obtained from previous clinical diagnosis at Wujin Hospital Affiliated with Jiangsu University in Changzhou City, China, which had been granted ethical review for the project, No. 2025-SR-018. Serum was separated from samples by centrifugation and stored at −80 °C until analysis. The baseline characteristics of MASLD patients and controls are shown in Additional file 2: Supplementary Table 4.

### ELISA

Serum levels of ATP6V1B2 were determined by ELISA using the ATP6V1B2 Assay Kit (AF0336-HA, Aifang Bio, China) according to the manufacturer’s instructions.

### Immunohistochemistry

Paraffin sections of liver tissue were first deparaffinized and rehydrated, and endogenous peroxidase was inactivated using 3%-H2O2 immersion for 20 min. Antigen repair was performed with citrate buffer (10 mM citrate buffer) and incubated for 1 hour with a drop of 5% BSA blocking solution. Liver tissue sections were incubated with antibody ATP6V1B2 (sc-166122, Santa Cruz, USA) at 4 °C overnight. Subsequently, sections were incubated with biotin-labeled goat anti-mouse/rabbit IgG and streptavidin-peroxidase complex (SA1020, BOSTER, China). Finally, liver tissue sections were stained using DAB horseradish peroxidase chromogenic kit (AR1022, BOSTER, China) and cell nuclei were stained with hematoxylin. The field of view was captured randomly using a pathology section scanner (InteMedic, Guangzhou, China).

### Hematoxylin and Eosin (H&E) staining

Liver tissue samples were fixed with 4% paraformaldehyde, embedded in paraffin and sectioned. The paraffin sections were deparaffinized and rehydrated, and then HE staining was performed to observe the degree of liver tissue damage under the microscope.

### cDNA and siRNA transfection

ATP6V1B2 knockdown plasmids and viruses were constructed by Human Fenghui Biotechnology Co., Ltd. (Hunan, China). Overexpression plasmids were purchased from Vigen Biotech (Zhenjiang, China) and transfected into HepG2 cells. ATP6V1B2 siRNA and its sequence (Additional file 2: Table S3) were purchased from GenePharma Co., Ltd. (Shanghai, China). Cells were transiently transfected using Lipofectamine 2000 reagent (Invitrogen, USA; Cat. No. 11668500) according to the manufacturer’s instructions. Cells were harvested 48 hours after transfection for subsequent experiments.

### mRFP-GFP-LC3B transfection

HepG2 cells were infected using GFP-mRFP-LC3 lentiviral titer samples (Vigen Biotech, China), and after 48–72 h of lentiviral infection, when the infection efficiency was around 80% and the cell confluence was at 60–70%, puromycin was added at a final concentration of 1 µg/ml to screen for stable cell lines.

### Lysosomal staining and analysis

Lysosomal activity in HepG2 cells was analyzed using lysosomal red fluorescent probe staining. Using LysoTracker Red (C1046, Beyotime, China) probe diluted at 1:5000 in serum-free medium, cells were added and incubated for 40 min at 37 °C in the dark. After washing with PBS, the cells were fixed with glutaraldehyde (PH9003, Feijin Research, China) and then stained with Hoechst 33342 staining solution (C1026, Beyotime, China) for nuclei, and the red lysosomes in the cells were observed by fluorescence microscopy (Olympus, Japan).

### Mitochondrial membrane potential assay

JC-1 fluorescent probe (C2003S, Beyotime, China) was diluted in serum-free medium at a ratio of 1:1000, and then cells were added and incubated in the dark at 37 °C for 20 min. Cells were fixed using 4% paraformaldehyde, and nuclei were stained with Hoechst 33342 (C1026, Beyotime, China). Fluorescent images were captured using a fluorescence microscope (Olympus, Japan) in a randomly selected field of view.

### Oil red O staining

Oil red O powder was prepared into 0.5% oil red O (O0625-25G, Sigma, USA) solution using isopropanol, and the staining solution was diluted and filtered at a ratio of 3:2 oil red O solution and distilled water. After OPA treatment, the cells were first fixed using 4% paraformaldehyde, and then the diluted oil red O staining solution was added and stained for 20 min, followed by immersion in 60% isopropyl alcohol for 30 s to remove the background. The nuclei were stained with hematoxylin staining solution (BP-DL004, Sbjbio, China), and the distribution of cellular oil droplets was observed by microscope (Olympus, Japan).

### Nile Red staining

1 mg of Nile Red powder (72485, Sigma, USA) was dissolved in 1 ml of 100% acetone prepared as 1 mg/mL stock solution. After OPA treatment, cells were stained with 1:3000 diluted fluorescent Nile Red working solution for 3 min at room temperature, washed with PBS, and then nuclei were stained using Hoechst 33342 (C1026, Beyotime, China). The stained cells were subsequently analyzed using a fluorescence microscope (Olympus, Japan).

### BODIPY staining

Lipid droplets were observed using the Lipid Droplet Green Fluorescence Detection Kit (C2053S, Beyotime, China), cells were first fixed using 4% paraformaldehyde, washed with PBS, a 1:1000 dilution of Bodipy formulated as Staining Solution was added to the cells, and the cells were incubated for 15 min at room temperature, washed with PBS, and then the nuclei were stained using Hoechst 33342 (C1026, Beyotime, China) to stain the nuclei. The stained cells were subsequently analyzed using a fluorescence microscope (Olympus, Japan).

### ROS assay

Reactive oxygen species (ROS) were detected using the Reactive Oxygen Species Detection Kit (S0033S, Beyotime, China). DCFH-DA was diluted in PBS at a ratio of 1:1000, added to the cells, and incubated for 20 minutes at 37 °C in a cell culture incubator. After incubation, the unincorporated DCFH-DA was removed by washing with PBS. The nuclei of the cells were stained with Hoechst 33342 (C1026, Beyotime, China) to visualize the cell nuclei. Fluorescence images were captured using a fluorescence microscope (Olympus, Japan) from randomly selected fields of view.

### MDA, GSH and SOD assays

Cell samples were collected, homogenized, and centrifuged at 4 °C. Subsequently, the levels of malondialdehyde (MDA), reduced glutathione (GSH), and total superoxide dismutase (SOD) in the cell samples were detected using the Lipid Peroxidation MDA Assay Kit (S0131S, Beyotime, China), Reduced Glutathione (GSH) Assay Kit (A006-2-1, Nanjing Jiancheng Bioengineering Institute, China), and Total SOD Activity Assay Kit (S0109, Beyotime, China), respectively.

### DQ-Red BSA staining

Prepare a solution of DQ-Red BSA (HY-D2449, MCE, USA) at a working concentration of 10 µg/ml. Add the required amount of DQ-Red BSA to the culture medium and incubate at 37 °C for 5 minutes. After washing the cell culture plate with PBS, add the DQ-Red BSA working solution and incubate in a cell culture incubator for 6 hours. At the desired time point, fix the cells with 4% paraformaldehyde for 15 minutes and stain with Hoechst 33342 staining solution for 5 minutes. Images are captured using a fluorescence microscope.

### Statistical analysis

For statistical analysis, we utilized SPSS 19.0 statistical software and GraphPad Prism 9 software to analyze the data. Chi-square tests and independent sample *t*-tests were used to compare the baseline characteristics of MASLD patients and controls. If the data followed a normal distribution (i.e., parameterized data), it was presented as mean ± standard deviation (SD). Before conducting the Student’s t-test to determine the statistical significance between two groups, we performed Levene’s test for equality of variances. Student’s *t*-test was utilized under the assumption that the data originates from a normal distribution and exhibits homogeneity of variance. For studies involving more than two groups, one-way analyses of variance (ANOVA) were conducted. All statistical tests were performed using a two-tailed approach. For the level of significance, we adopted a *p*-value of less than 0.05 as the standard for statistical significance.

## Supplementary information

Supplementary figure legends

Supplementary information

Supplementary figure 1

Supplementary figure 2

Supplementary figure 3

Supplementary figure 4

Western blot repetitions

## Data Availability

All data generated or used during the study appear in the submitted article.
