# Supplementary material for: ATP6V1B2 alleviates hepatic steatosis by promoting lysosomal acidification in hepatocytes
Source: Cell Death Discov. 2026 Mar 24;12:170. doi: 10.1038/s41420-026-03052-8 (PMC13040012; doi:10.1038/s41420-026-03052-8)
Supplement: Supplementary file 1 — Supplementary figure legends [file 41420_2026_3052_MOESM1_ESM.docx]

**Supplementary Fig. 1 OPA increases cellular lipid deposition and protein outcome quantification.**

**A** HE staining results of liver tissue sections of NCD-fed, HFD-fed, or MCD-fed mice. (Scale bar = 50 μm). **B** Quantification of ATP6V1B2 protein expression levels in the livers of NCD-fed or HFD-fed mice. **C** Quantification of ATP6V1B2 protein expression levels in the livers of NCD-fed or MCD-fed mice. **D** Nile red staining of cellular lipid droplets of HepG2 cells treated with OPA for 24 hours. (Scale bar = 50 μm). **E** Bodipy probe staining of lipid droplets of MPH treated with OPA for 24 hours. (Scale bar = 20 μm). **F** Quantification of ATP6V1B2 protein expression levels in HepG2 cells after OPA treatment. **G** Quantification of ATP6V1B2 protein expression levels in MPH after OPA treatment. **H** Quantification of immunofluorescence of HepG2 cells treated with OPA. **I** Quantification of immunofluorescence of MPH treated with OPA. All data were expressed as the means ± SD of at least 3 independent experiments, ** P* < 0.05; ***P* < 0.01; ****P* < 0.001

**Supplementary Fig. 2 OPA activates the mTOR signaling pathway and induces TFEB degradation in lipotoxic hepatocytes**. **A** Western blot analysis of ATP6V1B2, mTOR, p-mTOR, TFEB, and p-TFEB protein expression and quantitative analysis in HepG2 cells 24 hours after OPA treatment. **B.** Immunofluorescence staining of TFEB in HepG2 cells 24 hours after OPA treatment. (Scale bar = 20 μm). All data were expressed as the means ± SD of at least 3 independent experiments, ** P* < 0.05; ***P* < 0.01; ****P* < 0.001

**Supplementary Fig. 3 Knockdown of ATP6V1B2 induces ER stress in HepG2 cells. A** Western blot detection of ATP6V1B2 protein expression in HepG2 cells transfected with siCtr and siATP6V1B2. **B** Western blot detection of ATP6V1B2 protein expression in HepG2-shCtr and HepG2-shATP6V1B2. **C** Quantification of mRNA expression levels of ATP6V1B2 in HepG2 cells transfected with shCtr and shATP6V1B2 plasmids (1 µg and 2 µg). **D** Quantification of mRNA expression levels of ER stress markers GRP78, CHOP, PERK, IRE1, and XBP1 in HepG2-shCtr and HepG2-shATP6V1B2 cells. **E** Western blot detection of the protein expression levels and quantification of the ER stress markers GRP78, CHOP, PERK, IRE1, and XBP1 in HepG2-shCtr and HepG2-shATP6V1B2 cells. All data were expressed as the means ± SD of at least 3 independent experiments, ** P* < 0.05; ***P* < 0.01; ****P* < 0.001

**Supplementary Fig. 4 Overexpression of ATP6V1B2 alleviates ER stress and inflammatory responses in HepG2 cells. A** Quantification of mRNA levels of ATP6V1B2, TGFβ, and IL-8 in HepG2 cells transfected with pCtr and pATP6V1B2 plasmids (0.5 g and 1 µg). **B** Western blot detection and quantification of the protein expression levels of ER stress markers GRP78, CHOP, PERK, IRE1, and XBP1 in OPA-treated HepG2 cells transfected with control plasmid (pCtr) or pATP6V1B2 plasmid (0.5 µg or 1 µg). **C** HepG2-shCtr and HepG2-shATP6V1B2 were stained with DQ-BSA for 6 hours. (Scale bar = 20 µm). All data were expressed as the means ± SD of at least 3 independent experiments, ** P* < 0.05; ***P* < 0.01; ****P* < 0.001
