# Supplementary material for: ATP6V1B2 alleviates hepatic steatosis by promoting lysosomal acidification in hepatocytes
Source: Cell Death Discov. 2026 Mar 24;12:170. doi: 10.1038/s41420-026-03052-8 (PMC13040012; doi:10.1038/s41420-026-03052-8)
Supplement: Supplementary file 2 — Supplementary information [file 41420_2026_3052_MOESM2_ESM.docx]

Supplementary information

**Supplementary Table 1. Summary of primary antibodies used in Western Blot, Immunochemistry, immunofluorescence**

| **Antibody** | **for WB** | **for IHC** | **for IF** | **Cat. No.** | **Company** |
| --- | --- | --- | --- | --- | --- |
| ATP6V1B2 |  | 1:200 | 1:100 | sc-166122 | Santa Cruz |
| ATP6V1B2 | 1:5000 | 1:200 | 1:200 | 15097-1-AP | Proteintech |
| GAPDH | 1:5000 |  |  | AC002 | Abclonal |
| LAMP1 | 1:500 |  |  | sc-20011 | Santa Cruz |
| LAMP1 |  |  | 1:200 | 67300-1-Ig | proteintech |
| FASN | 1:500 |  | 1:200 | sc-48357 | Santa Cruz |
| LC3B | 1:500 |  | 1:100 | A19665 | Abclonal |
| P62 | 1:5000 |  |  | 18420-1-AP | Proteintech |
| TFEB | 1:2000 |  | 1:100 | 13372-1-AP | Proteintech |
| p-TFEB | 1:1000 |  |  | TA3708 | ABMART |
| mTOR | 1:5000 |  |  | 66888-1-IG | Proteintech |
| p-mTOR | 1:1000 |  |  | T56571 | ABMART |
| IRE1 | 1:1000 |  |  | TA7651 | ABMART |
| XBP1 | 1:2000 |  |  | 24868-1-AP | Proteintech |
| GPR78 | 1:1000 |  |  | P23089-2 | ABMART |
| CHOP | 1:1000 |  |  | T56694 | ABMART |
| PERK | 1:1000 |  |  | TP52759 | ABMART |
| HRP Goat Anti-Rabbit IgG (H+L) | 1:5000 |  |  | AS014 | Abclonal |
| HRP Goat Anti-Mouse IgG (H+L) | 1:5000 |  |  | AS003 | Abclonal |
| Cy3-Goat Anti-Mouse IgG (H+L) |  |  | 1:200 | AS008 | Abclonal |
| FITC-Goat Anti-Rabbit IgG (H+L) |  |  | 1:200 | AS011 | Abclonal |
| Cy3 Goat Anti-Rabbit IgG (H+L) |  |  | 1:200 | AS007 | Abclonal |
| FITC Goat Anti-Mouse IgG (H+L) |  |  | 1:200 | AS001 | Abclonal |

**Supplementary Table 2. Primers in this study.**

| **Identifier** | **Forward (5’-3’)** | **Reverse (5’-3’)** |
| --- | --- | --- |
| **For qPCR** |  |  |
| **Homo-ATP6V1B** | TTGACCTTACCGGATGGCAC | CAAGGAAGTCTTCGGCCAGT |
| **Homo-β-Actin** | GACCTGTACGCCAACACAGT | GATAAGCCGTGGTTCTGGTC |
| **Homo-TGF-β** | TGATGTCACCGGAGTTGTGC | CGGTAGTGAACCCGTTGATG |
| **Homo-IL-8** | CCAGGAAGAAACCACCGGA | GAAATCAGGAAGGCTGCCAAG |
| **Mus-ATP6V1B2** | ATGCCATCGGTAAGGACGTG | GATTCGAAGCAACTGCCAGC |
| **Mus-β-Actin** | ATGCCCTGAGGCTCTTTTCC | CAGCTCAGTAACAGTCCGCC |
| **Homo- IRE1** | CCATGCCGAAGTTCAGATGG | GTGAGGCCGCATAGTCAAAG |
| **Homo- XBP1** | CCGGAGCTGGGTATCTCAAA | GGCAAAAGTGTCCTCCCAAG |
| **Homo- GRP78** | CGGTCTACTATGAAGCCCGT | CATCTGGGTTTATGCCACGG |
| **Homo- CHOP** | TCTTGACCCTGCTTCTCTGG | GCTGTGCCACTTTCCTTTCA |
| **Homo- PERK** | GTCCGGAACCAGACGATGAG | CCCACTGCTTTTTTACCATGATTT |

**Supplementary Table 3. RNA oligo in this study.**

| Identifier | Sense | Antisense |
| --- | --- | --- |
| **For Knockdown** | | |
| ATP6V1B2 oligo | GCUGGUUUGGUAAAGAAAUTT | AUUUCUUUACCAAACCAGCTT |
| Control oligo | UUCUCCGAACGUGUCACGUTT | ACGUGACACGUUCGGAGAATT |

**Supplementary Table 4. Clinical Data Table of Baseline Characteristics**

| Characteristics | MASLD  (n=58) | Controls  (n=66) | P value |
| --- | --- | --- | --- |
| Gender |  |  | 0.237 |
| Female | 36 (29%) | 34 (27.4%) |  |
| Male | 22 (17.7%) | 32 (25.8%) |  |
| Age, median (IQR) | 35 (30, 49) | 35 (27.25, 39.75) | 0.126 |
| BIM, median (IQR) | 23.882 (23.3, 24.7) | 23.588 (23.1, 24.4) | 0.053 |

Abbreviations: BMI, body mass index
