# Supplementary figures and images for: ATP6V1B2 alleviates hepatic steatosis by promoting lysosomal acidification in hepatocytes
Source: Cell Death Discov. 2026 Mar 24;12:170. doi: 10.1038/s41420-026-03052-8 (PMC13040012; doi:10.1038/s41420-026-03052-8)

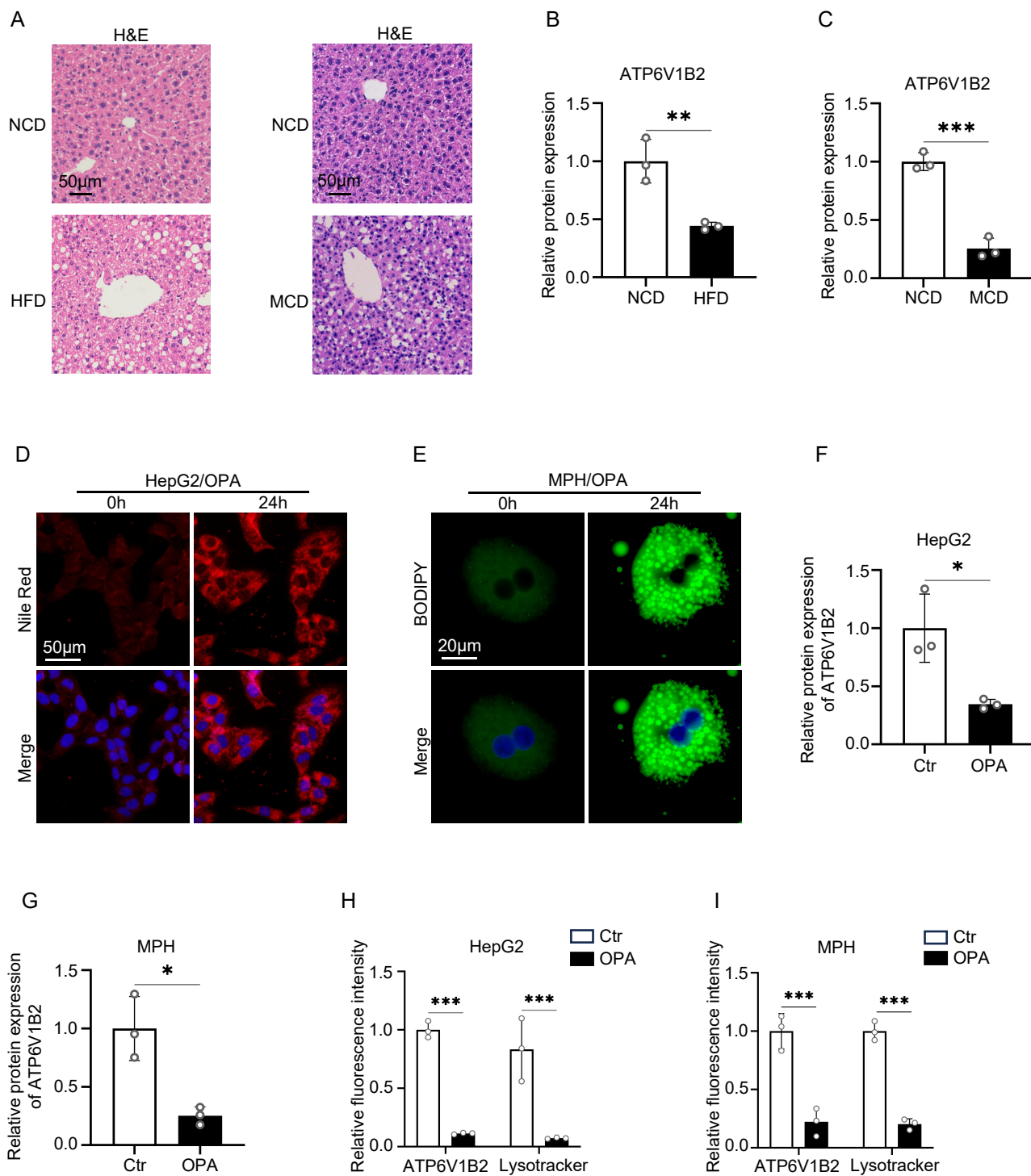

Supplement: Supplementary file 3 — Supplementary figure 1 [file 41420_2026_3052_MOESM3_ESM.pdf]

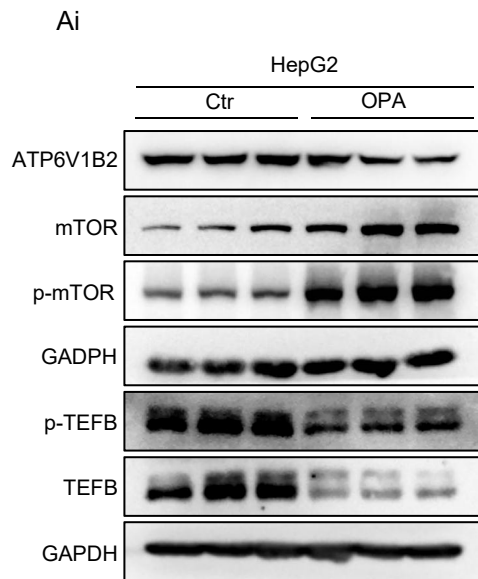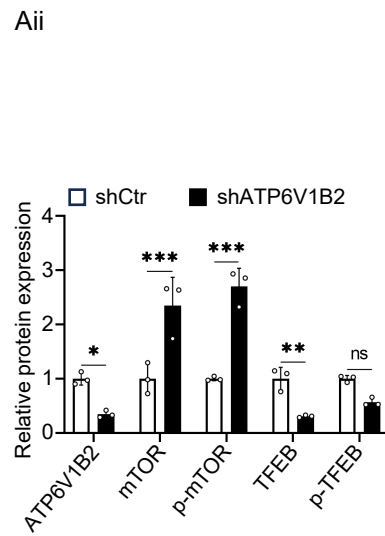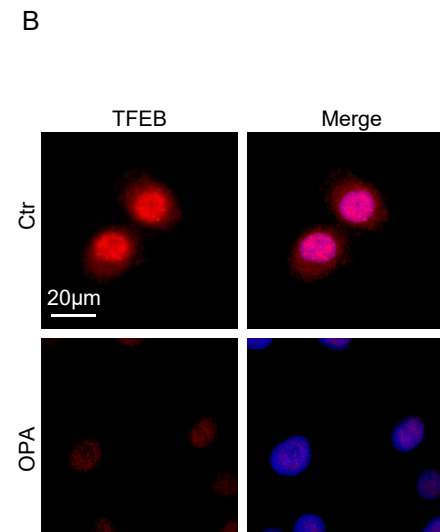

Supplement: Supplementary file 4 — Supplementary figure 2 [file 41420_2026_3052_MOESM4_ESM.pdf]

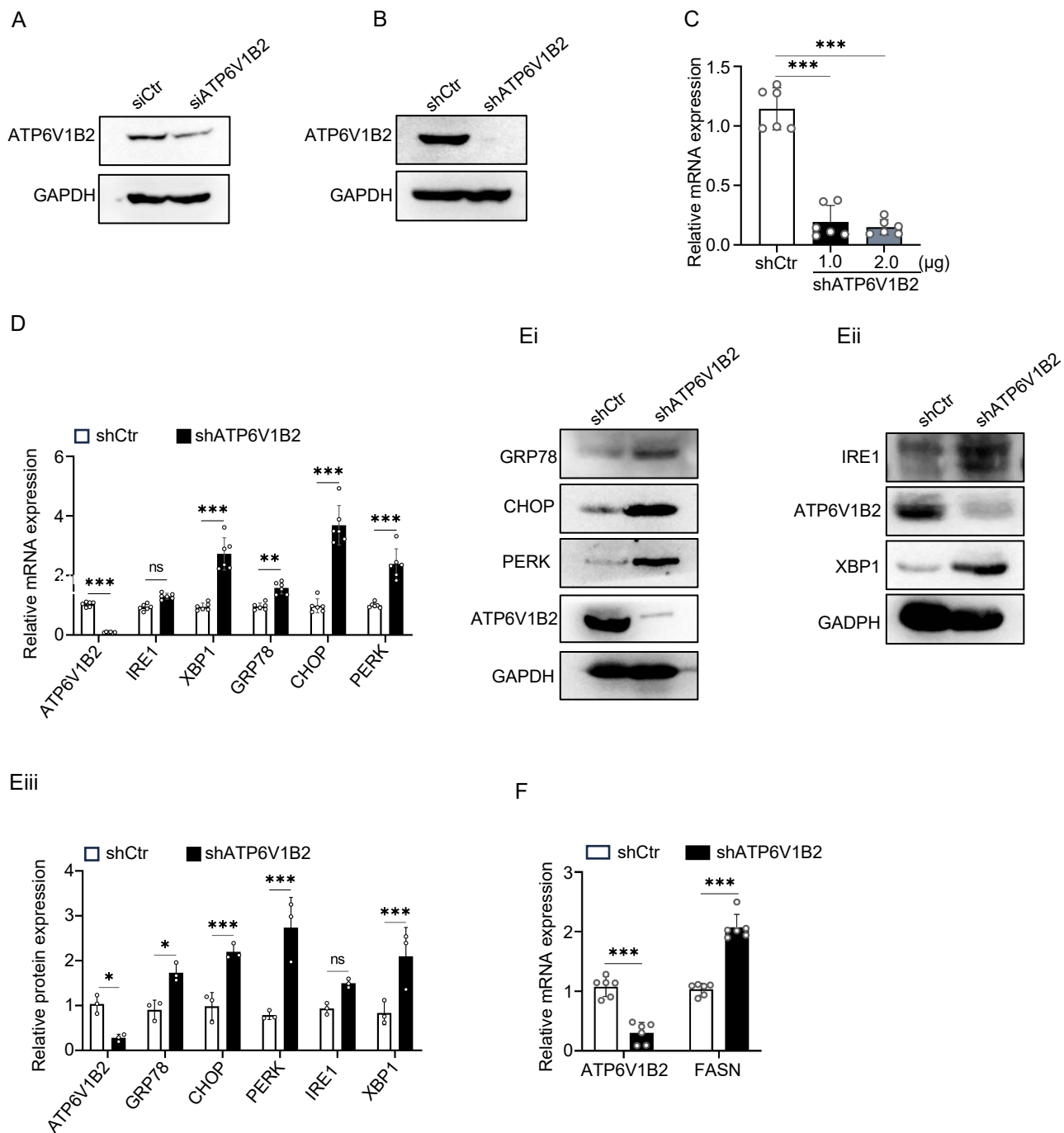

Supplement: Supplementary file 5 — Supplementary figure 3 [file 41420_2026_3052_MOESM5_ESM.pdf]

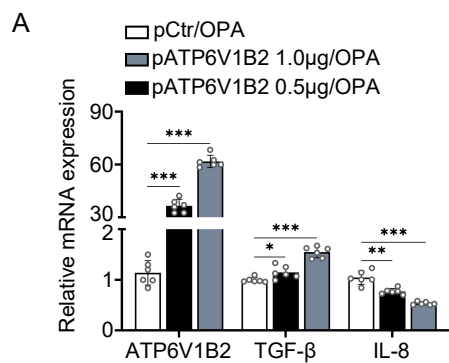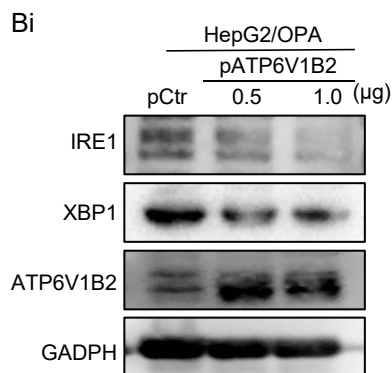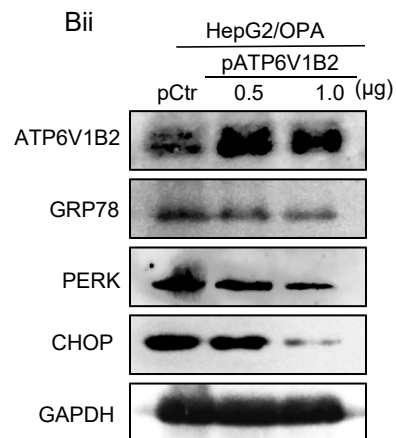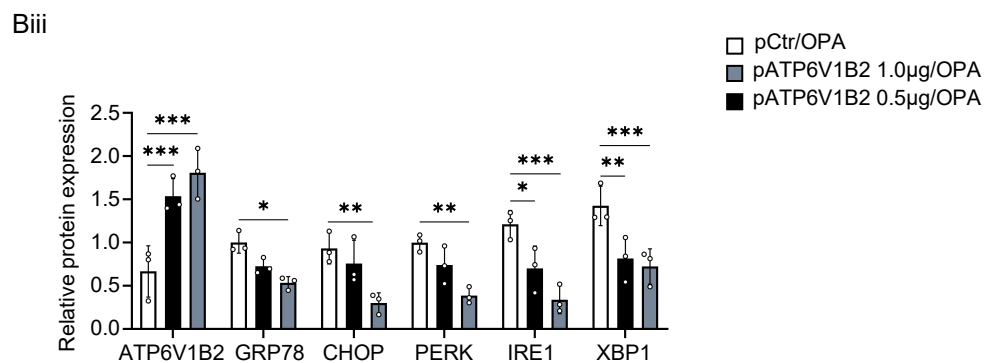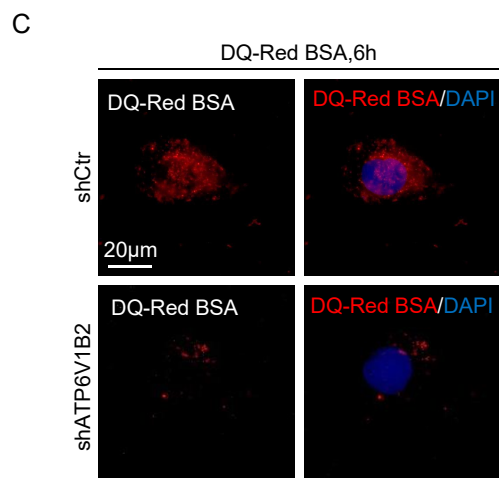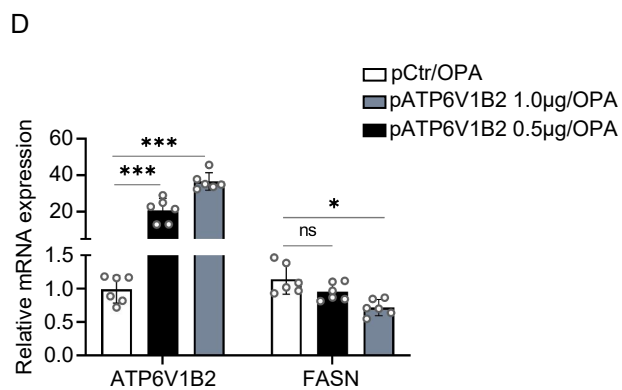

Supplement: Supplementary file 6 — Supplementary figure 4 [file 41420_2026_3052_MOESM6_ESM.pdf]
