# Supplementary material for: ATP6V1B2 alleviates hepatic steatosis by promoting lysosomal acidification in hepatocytes
Source: Cell Death Discov. 2026 Mar 24;12:170. doi: 10.1038/s41420-026-03052-8 (PMC13040012; doi:10.1038/s41420-026-03052-8)

Fig.2E

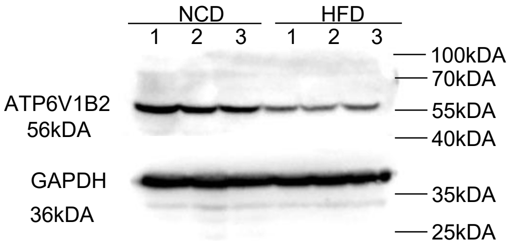

Fig.2G

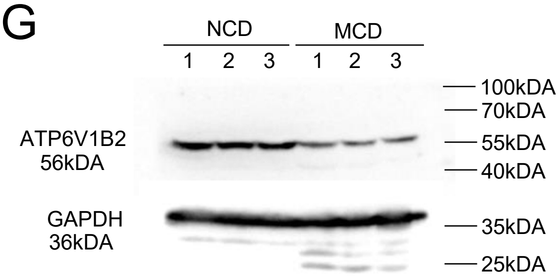

Fig.2I

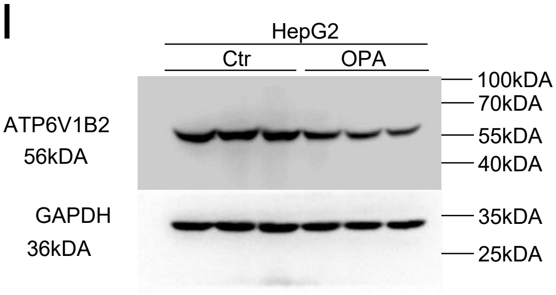

Fig.2K

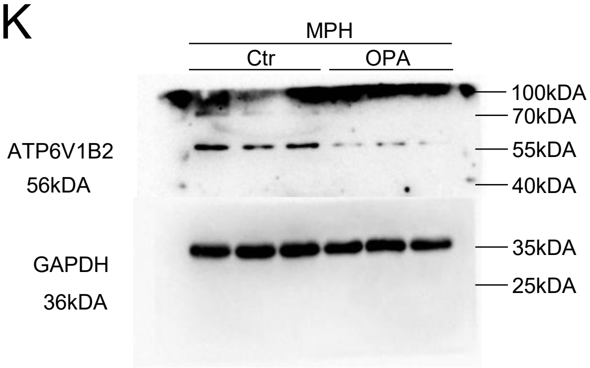

Fig.3A

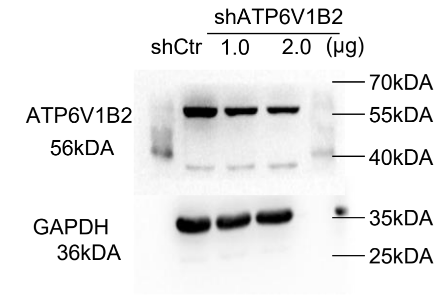
shATP6V1B2
shCtr
1.0
2.0
(μg)
ATP6V1B2
56kDA
GAPDH
36kDA
70kDA
55kDA
40kDA
35kDA
25kDA

Fig.3J

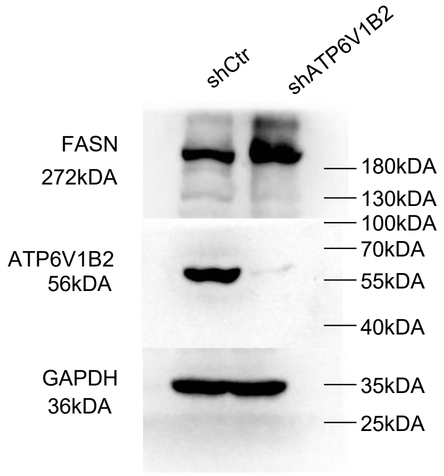
shCtr
shATP6V1B2
FASN
272kDA
ATP6V1B2
56kDA
GAPDH
36kDA
180kDA
130kDA
100kDA
70kDA
55kDA
40kDA
35kDA
25kDA

Fig.4A

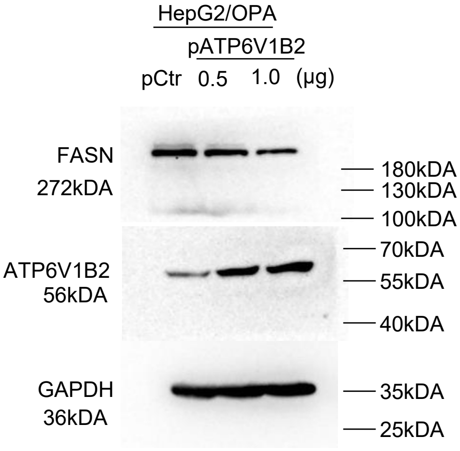
HepG2/OPA
pATP6V1B2
pCtr 0.5 1.0 (μg)
FASN
272kDA
ATP6V1B2
56kDA
GAPDH
36kDA
180kDA
130kDA
100kDA
70kDA
55kDA
40kDA
35kDA
25kDA

Fig.4H

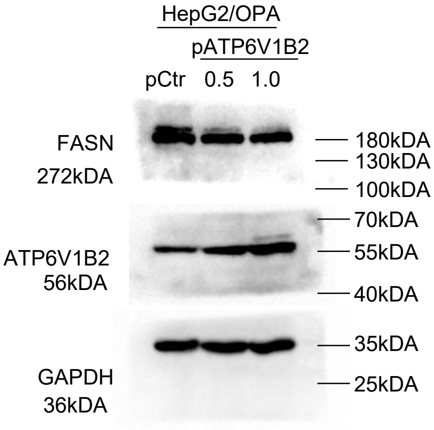
HepG2/OPA
pATP6V1B2
pCtr 0.5 1.0
FASN
272kDA
ATP6V1B2
56kDA
GAPDH
36kDA
180kDA
130kDA
100kDA
70kDA
55kDA
40kDA
35kDA
25kDA

Fig.5A

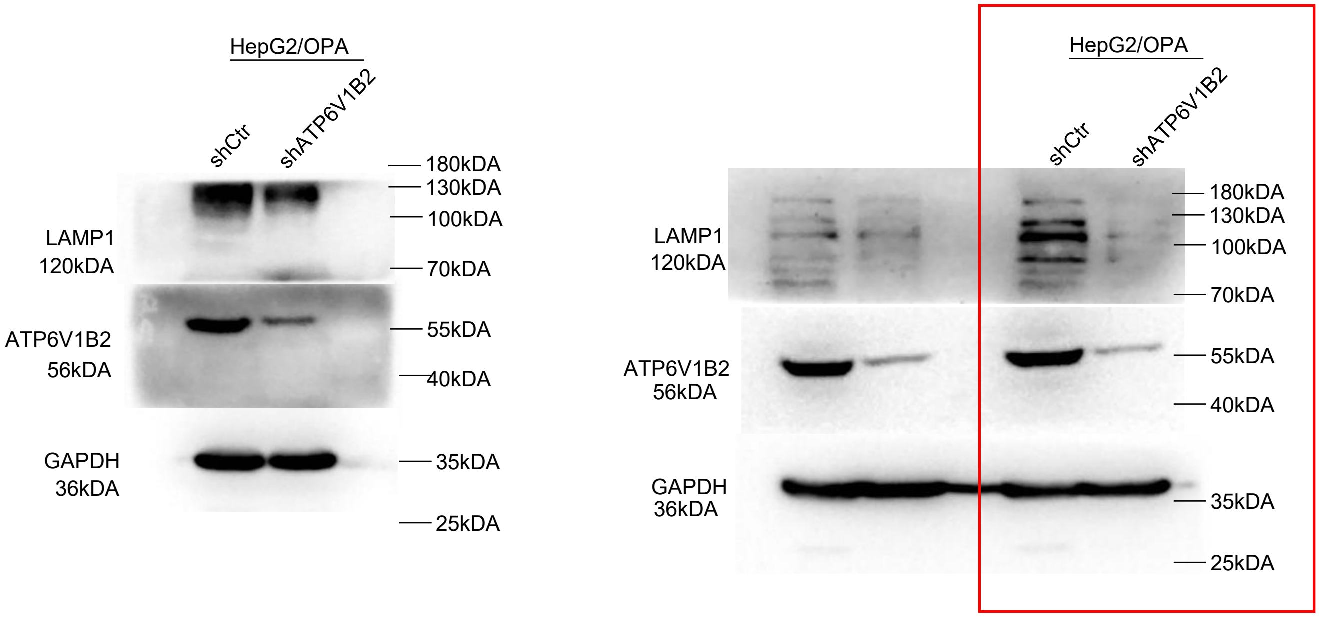
HepG2/OPA
shCtr
shATP6V1B2
LAMP1
120kDA
ATP6V1B2
56kDA
GAPDH
36kDA
180kDA
130kDA
100kDA
70kDA
55kDA
40kDA
35kDA
25kDA
HepG2/OPA
shCtr
shATP6V1B2
LAMP1
120kDA
ATP6V1B2
56kDA
GAPDH
36kDA
180kDA
130kDA
100kDA
70kDA
55kDA
40kDA
35kDA
25kDA

Fig.5C

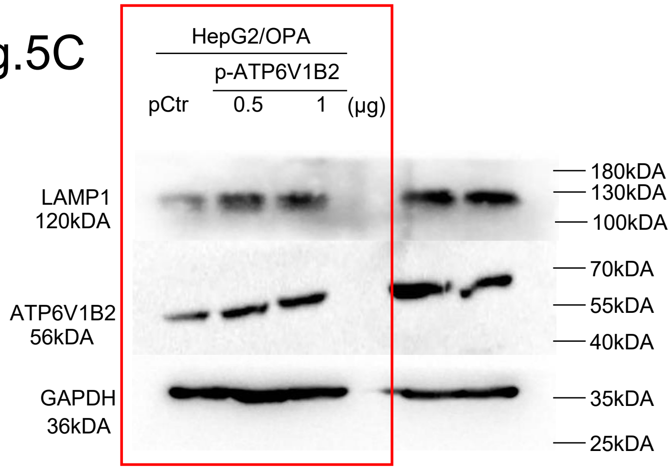
HepG2/OPA
p-ATP6V1B2
pCtr
0.5
1
(μg)
LAMP1
120kDA
ATP6V1B2
56kDA
GAPDH
36kDA
180kDA
130kDA
100kDA
70kDA
55kDA
40kDA
35kDA
25kDA

Fig.6A

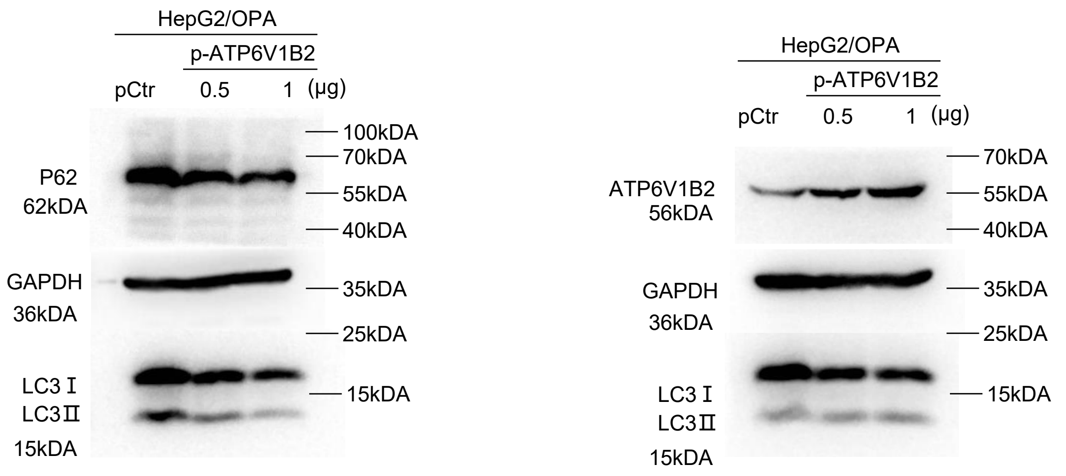
HepG2/OPA
p-ATP6V1B2
pCtr
0.5
1
(μg)
P62
62kDA
GAPDH
36kDA
LC3 I
LC3 II
15kDA
100kDA
70kDA
55kDA
40kDA
35kDA
25kDA
15kDA
HepG2/OPA
p-ATP6V1B2
pCtr
0.5
1
(μg)
ATP6V1B2
56kDA
GAPDH
36kDA
LC3 I
LC3 II
15kDA
70kDA
55kDA
40kDA
35kDA
25kDA
15kDA

Fig.6B

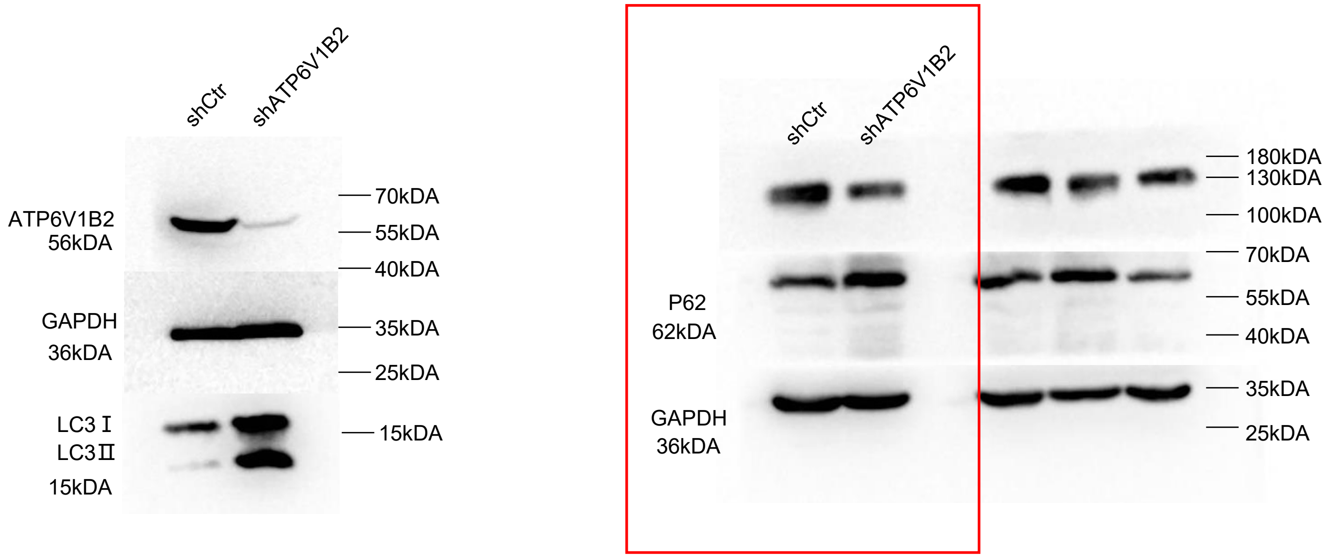
shCtr
shATP6V1B2
ATP6V1B2
56kDA
GAPDH
36kDA
LC3 I
LC3 II
15kDA
70kDA
55kDA
40kDA
35kDA
25kDA
15kDA
shCtr
shATP6V1B2
P62
62kDA
GAPDH
36kDA
180kDA
130kDA
100kDA
70kDA
55kDA
40kDA
35kDA
25kDA

Fig.6C

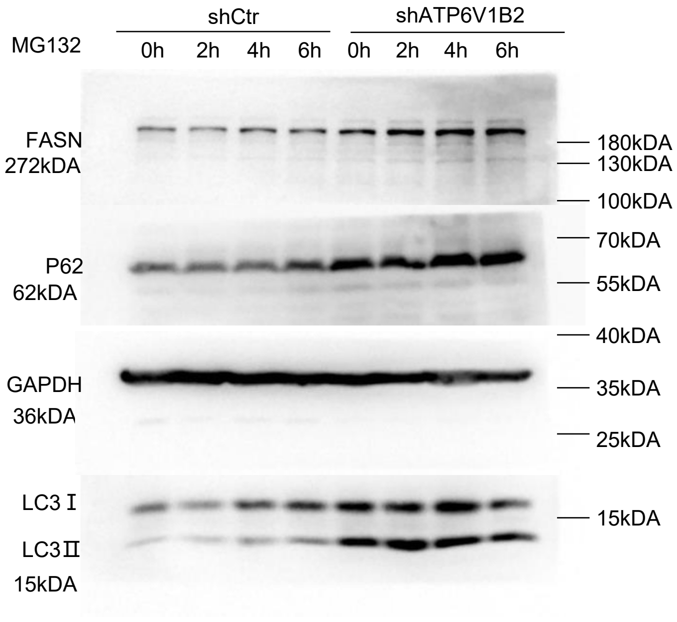
shCtr
shATP6V1B2
MG132
0h 2h 4h 6h 0h 2h 4h 6h
FASN
272kDA
P62
62kDA
GAPDH
36kDA
LC3 I
LC3 II
15kDA
180kDA
130kDA
100kDA
70kDA
55kDA
40kDA
35kDA
25kDA
15kDA

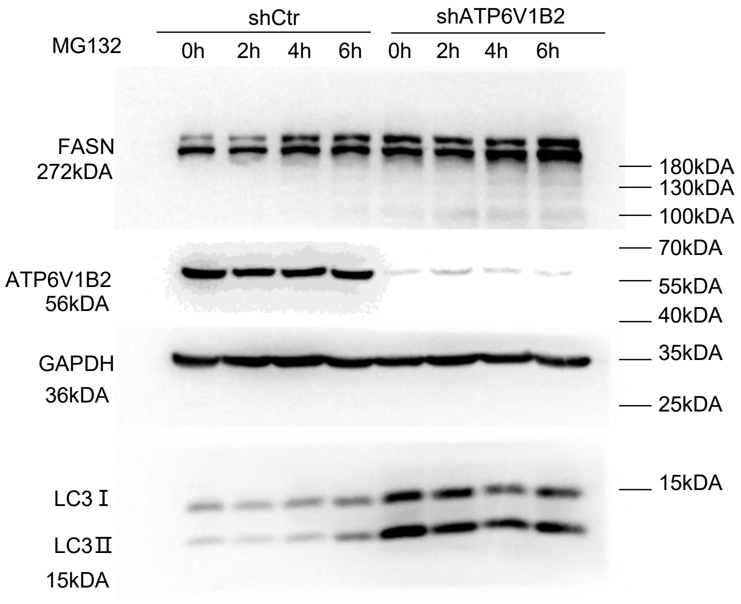
shCtr
shATP6V1B2
MG132
0h 2h 4h 6h 0h 2h 4h 6h
FASN
272kDA
ATP6V1B2
56kDA
GAPDH
36kDA
LC3 I
LC3 II
15kDA
180kDA
130kDA
100kDA
70kDA
55kDA
40kDA
35kDA
25kDA
15kDA

Fig.6D

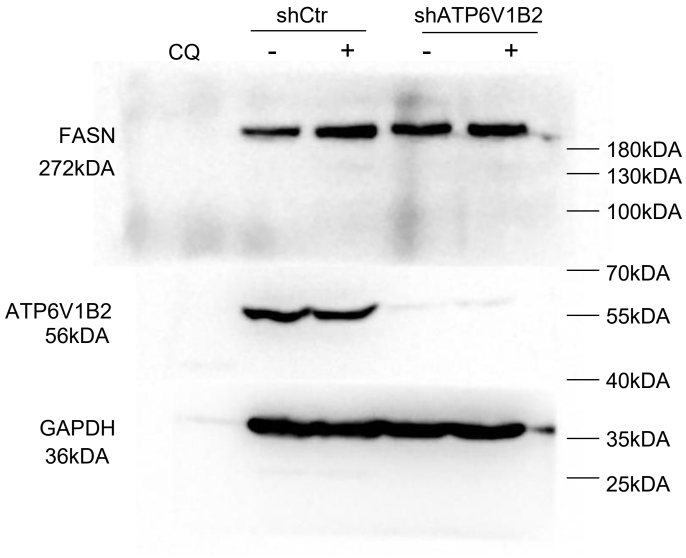
shCtr
shATP6V1B2
CQ
- + - +
FASN
272kDA
ATP6V1B2
56kDA
GAPDH
36kDA
180kDA
130kDA
100kDA
70kDA
55kDA
40kDA
35kDA
25kDA

Supplementary Fig. 2A

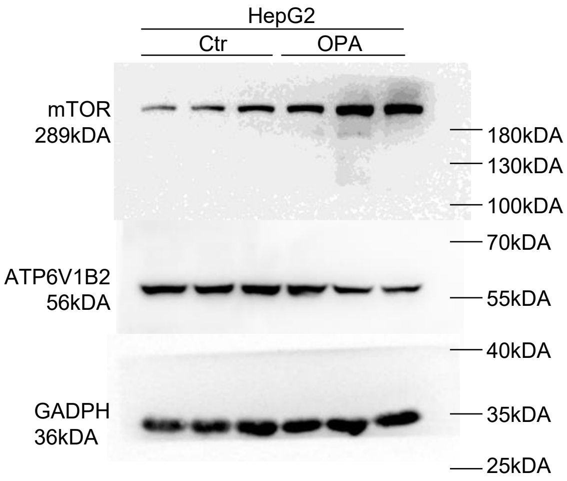

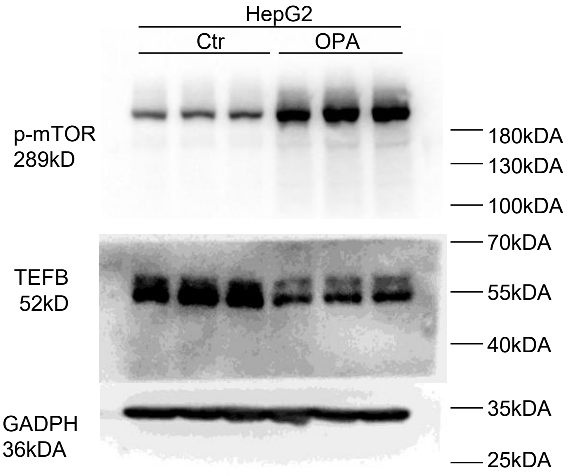

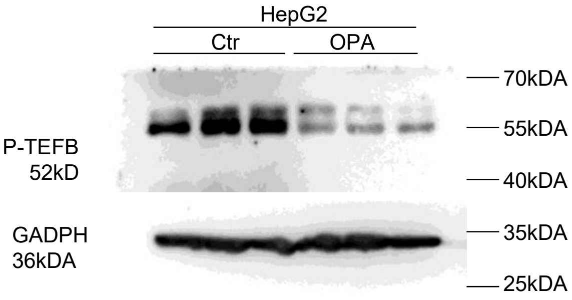

Supplementary Fig. 3A

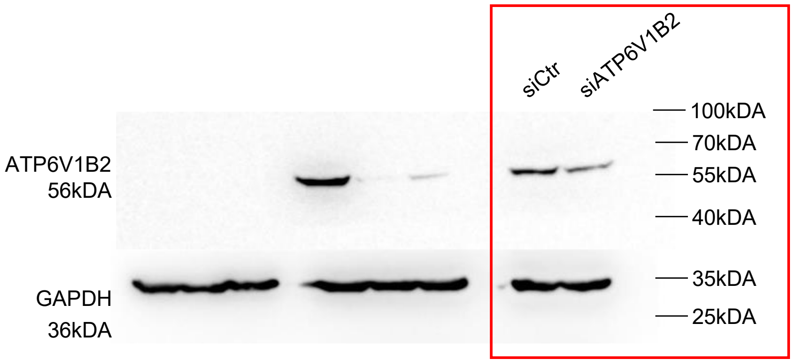

Supplementary Fig. 3B

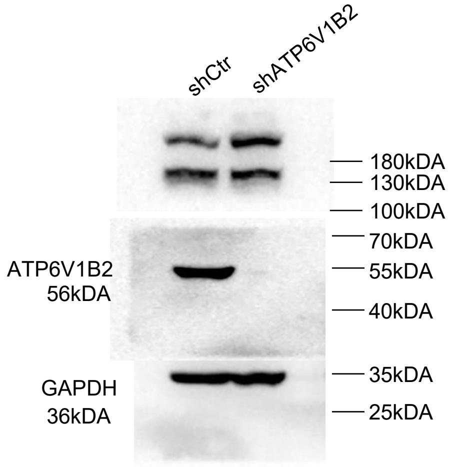

Supplementary Fig. 3E

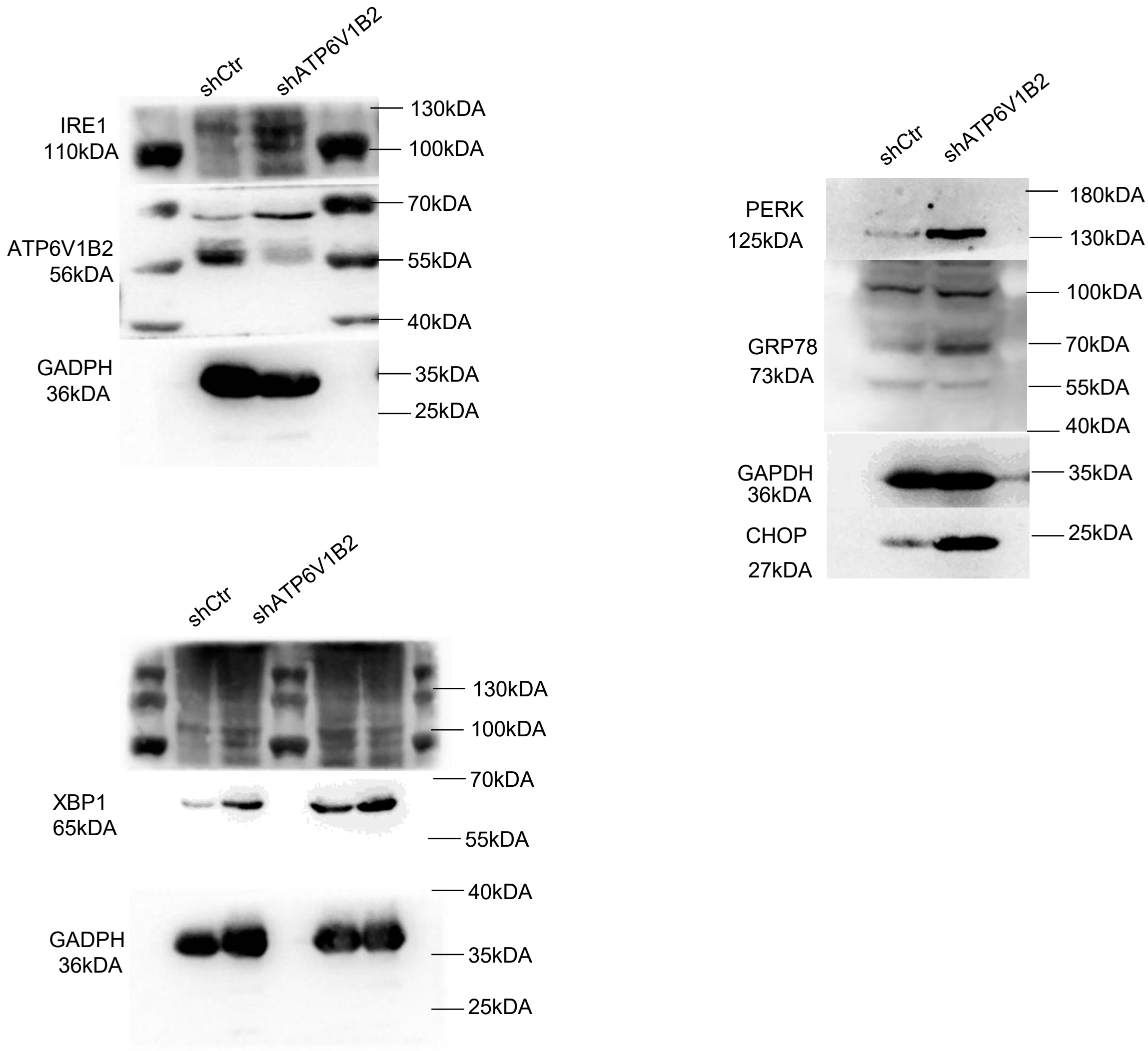

Supplementary Fig. 4B

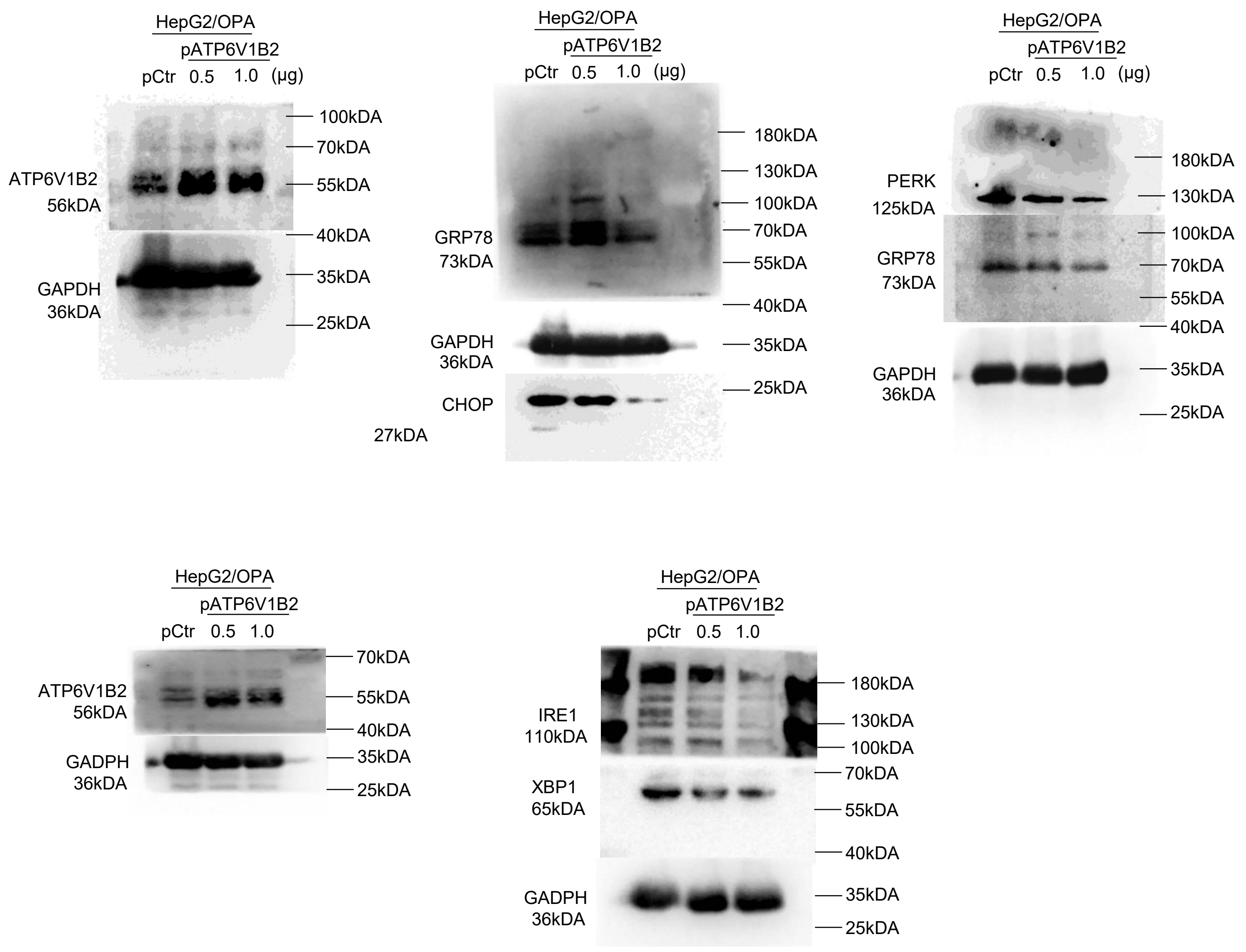

Supplement: Supplementary file 7 — Western blot repetitions [file 41420_2026_3052_MOESM7_ESM.pdf]
